# Palmitic acid‐ and cysteine‐functionalized nanoparticles overcome mucus and epithelial barrier for oral delivery of drug

**DOI:** 10.1002/btm2.10510

**Published:** 2023-03-23

**Authors:** Yinzhuo Xie, Zheng Jin, Da Ma, Tan Hui Yin, Kai Zhao

**Affiliations:** ^1^ Institute of Nanobiomaterials and Immunology, Zhejiang Provincial Key Laboratory of Plant Evolutionary Ecology and Conservation, School of Life Sciences, Taizhou University Taizhou 318000 China; ^2^ Key Laboratory of Microbiology, College of Heilongjiang Province, School of Life Sciences, Heilongjiang University Harbin 150080 China; ^3^ School of Pharmaceutical and Materials Engineering & Institute for Advanced Studies, Taizhou University Taizhou 318000 China; ^4^ Tunku Abdul Rahman University of Management and Technology Jalan Genting Kelang Kuala Lumpur 53300 Malaysia

**Keywords:** curcumin, mucus and epithelial barrier, oral delivery, palmitic acid and cysteine, quaternized chitosan‐based nanoparticles

## Abstract

Nanoparticles (NPs) used for oral administration have greatly improved drug bioavailability and therapeutic efficacy. Nevertheless, NPs are limited by biological barriers, such as gastrointestinal degradation, mucus barrier, and epithelial barrier. To solve these problems, we developed the PA‐N‐2‐HACC‐Cys NPs loaded with anti‐inflammatory hydrophobic drug curcumin (CUR) (CUR@PA‐N‐2‐HACC‐Cys NPs) by self‐assembled amphiphilic polymer, composed of the *N*‐2‐Hydroxypropyl trimethyl ammonium chloride chitosan (N‐2‐HACC), hydrophobic palmitic acid (PA), and cysteine (Cys). After oral administration, the CUR@PA‐N‐2‐HACC‐Cys NPs had good stability and sustained release under gastrointestinal conditions, followed by adhering to the intestine to achieve drug mucosal delivery. Additionally, the NPs could penetrate mucus and epithelial barriers to promote cellular uptake. The CUR@PA‐N‐2‐HACC‐Cys NPs could open tight junctions between cells for transepithelial transport while striking a balance between mucus interaction and diffusion through mucus. Notably, the CUR@PA‐N‐2‐HACC‐Cys NPs improved the oral bioavailability of CUR, which remarkably relieved colitis symptoms and promoted mucosal epithelial repair. Our findings proved that the CUR@PA‐N‐2‐HACC‐Cys NPs had excellent biocompatibility, could overcome mucus and epithelial barriers, and had significant application prospects for oral delivery of the hydrophobic drugs.

## 1 INTRODUCTION

Oral administration of drugs remains the most clinically practical route of administration due to its significance in improving patient comfort and compliance.^1^ However, the solubility and permeability of the drug affect the efficiency of drug absorption.^2^ Nanoparticle (NP)‐based drug delivery system (DDS) is developing as potential carriers to improve drug delivery and absorption.^3^ NPs function not only to protect drugs from enzymatic degradation and reduce side effects, but also significantly improve the oral bioavailability and treatment efficacy of drugs.^4^, ^5^, ^6^

NP‐based DDS need to pass through two physical barriers before entering systemic circulation. The first barrier is the mucus layers secreted by goblet cells,^7^ and the second is cellular uptake and transport across epithelial cells.^8^ As intestinal mucus is a strong barrier against foreign pathogens,^9^ it affected the efficacy of the oral delivery of NPs. Various strategies based on the hydrophilicity, shape, particle size, charge, and rigidity have been investigated to facilitate NP mucus penetration and mucoadhesive.^10^ NPs with neutral and hydrophilic surface properties exhibit good mucus permeability to overcome the mucus barrier,^11^ but hydrophilic/neutral surfaces also decrease the interaction with the cell membrane.^12^ In contrast, positively charged NPs can increase the residence time of drugs at absorption sites and facilitate cellular uptake through electrostatic interactions with negatively charged mucins and cell membranes.^13^ Notably, in addition to enhancing the mucoadhesion of NPs to prolong intestinal residence time, the balance between interaction with the mucus and diffusion through mucus should be considered to prevent the strong adhesion from removing the embedded NPs.^14^

However, even if the NPs successfully pass through the mucin steric barrier, epithelial cells can act as a physical barrier to hinder the uptake of the drug‐loaded NPs.^15^, ^16^ The epithelial barrier includes intestinal epithelium and cell junctions.^17^ It has been reported that NPs designed with a positively charged surface could enhance the uptake by epithelial cells,^18^ and hydrophobic surfaces are also preferable for efficient cellular internalization.^12^ Moreover, strategies of opening the tight junction (TJ) to facilitate transepithelial transport have also been widely studied to overcome epithelial barriers.^19^, ^20^

It is challenging to develop an oral DDS that can effectively overcome mucus and epithelial barriers. In recent years, much attention has been paid to cationic chitosan due to its excellent physicochemical properties, such as good biocompatibility, biodegradability, and mucoadhesion.^21^, ^22^ Furthermore, nano‐carriers developed using chitosan and its derivatives can reversibly open the epithelial TJ to promote intestinal drug absorption via transcellular and paracellular pathways.^23^ The poor solubility of chitosan limits the application of chitosan. However, amino and hydroxyl groups on chitosan chains are employed as substrates for various chemical reactions, allowing them to improve the properties of the initial polymer and introduce specific properties, such as mucoadhesion,^24^ antimicrobial activity,^25^ and solubility.^26^, ^27^ *N*‐2‐Hydroxypropyl trimethyl ammonium chloride chitosan (N‐2‐HACC), a quaternized chitosan derivative, exhibits excellent aqueous solubility over a wide pH range.^28^ Furthermore, due to the presence of quaternized amino groups, the N‐2‐HACC increases the residence time and drug concentration at absorption sites via strong electrostatic interaction with negatively charged mucins,^29^ thus improving the oral bioavailability of the drug.

In addition to positively charged surfaces, hydrophobicity of material surface is also preferred for cellular internalization.^30^ Amphiphilic polymers are macromolecular compounds containing hydrophilic and lipophilic segments.^31^ The amphiphilic polymer containing two incompatible parts with different solubility will tend to irreversibly equilibrate via self‐assembly in a selective solvent.^32^ NPs based on amphiphilic polymers can facilitate transport across the epithelium due to their surface mimicking the cellular phospholipid membrane.^33^ Palmitic acid (PA), a hexadecanoic saturated fatty acid, can be used as a hydrophobic chain donor to graft chitosan molecules, giving chitosan the amphiphilic structure and ability to self‐assemble micelles.^34^, ^35^ In addition, many permeation studies have shown that the permeation effect of polymers is further improved by immobilizing thiol groups. Cysteine (Cys) can significantly enhance the mucoadhesion and permeability of chitosan and its derivatives.^24^, ^36^ The excellent mucoadhesion property is mainly attributed to the covalent bonds formed between the thiol groups on Cys and the glycoproteins in the mucus, which is much stronger than the electrostatic interaction between chitosan and mucus layer.^37^, ^38^ Moreover, the thiol groups on Cys endow modified polymer with multifunctional group characteristics to achieve better drug loading. Therefore, it was possible to obtain an excellent nano‐carrier with the combined effect of PA, Cys, and N‐2‐HACC.

Curcumin (CUR) is a naturally hydrophobic polyphenol compound with excellent antioxidant, anti‐inflammatory, and anticancer activities.^39^, ^40^ Notably, CUR has a wide range of preventive properties against diseases, such as cardiovascular disease, various types of cancer, inflammation, and diabetes.^41^ Nonetheless, the practical application of CUR is greatly restricted owing to its physicochemical instability, high hydrophobicity, low bioavailability, and rapid systemic elimination.^42^, ^43^ Therefore, it is vital to design an efficient delivery system to improve the bioavailability of CUR.

In the study, we aimed to develop a NP‐based DDS that could effectively overcome mucus and epithelial barriers for the oral delivery of drug. The N‐2‐HACC could open the epithelial TJ to transport CUR on epithelial cells, and the polymerization of N‐2‐HACC with PA could enhance the hydrophobicity of N‐2‐HACC, thus contributing to cellular internalization, and endow N‐2‐HACC with self‐assembly into micelles, encapsulating CUR. The thiolated polymer (PA‐N‐2‐HACC‐Cys) has the potential to penetrate the mucus layer. In the present study, we investigated the stability, the interaction between NPs and mucus, and transepithelial transport efficiencies in mucus‐secreting cell models. The intestinal absorption in vivo and pharmacokinetics of NPs loaded with CUR after oral administration were also assessed. Finally, we investigated the therapeutic effect of CUR@PA‐N‐2‐HACC‐Cys NPs in mice with colitis. It was hypothesized that the PA‐ and Cys‐functionalized NPs could improve the hydrophilicity, stability, and bioavailability of drug in the gastrointestinal tract. Therefore, the strategies for functionalizing natural polymers open up a new avenue to the synthesis and formulation of NPs with good biocompatibility and multifunctional properties.

## 2 RESULTS AND DISCUSSION

### 2.1 Characterization of the PA‐N‐2‐HACC‐Cys

**FIGURE 1:**
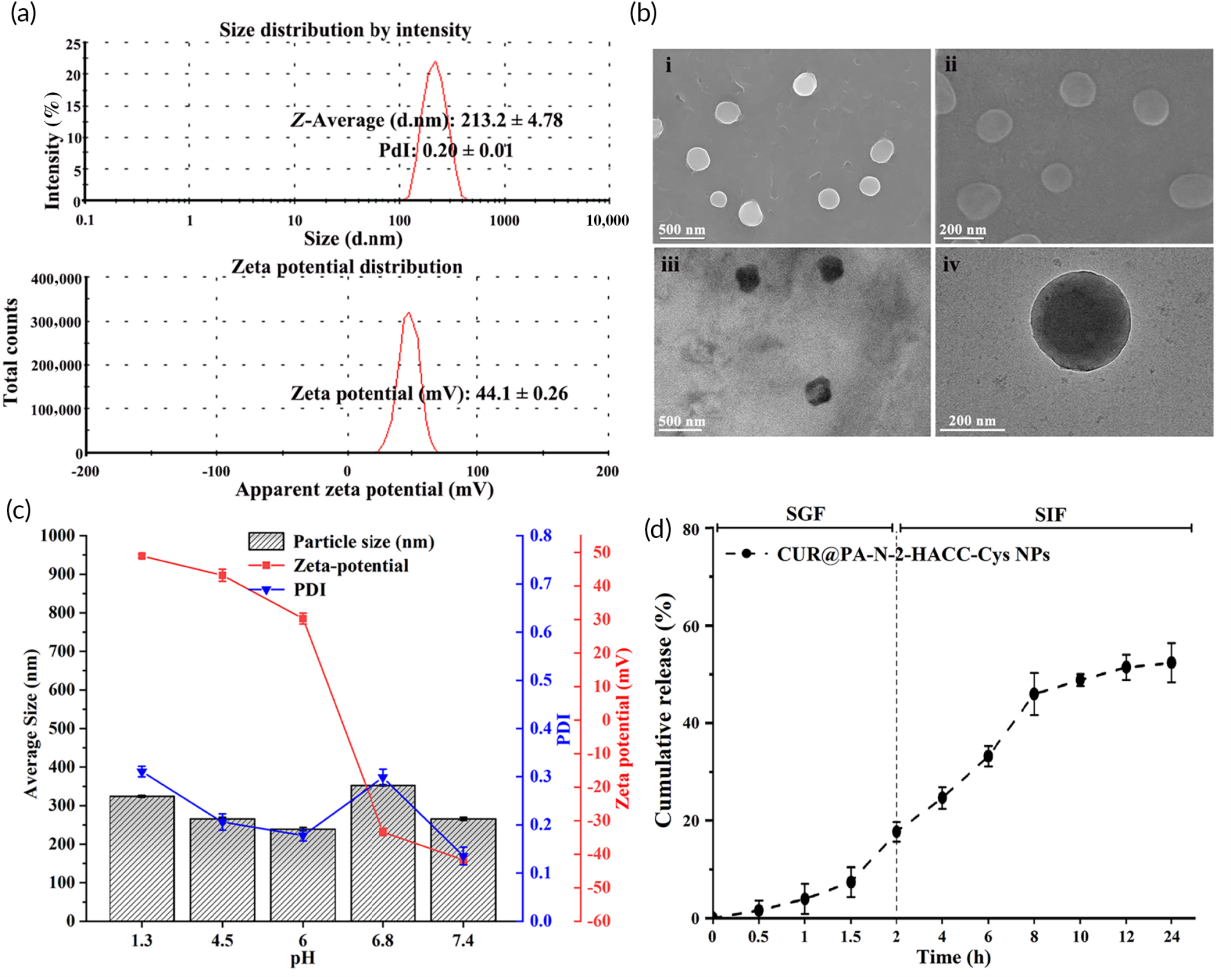
Characterization of the CUR@PA‐N‐2‐HACC‐Cys NPs (*n* = 3). (a) Diagram of particle size and zeta potential; (b) SEM and TEM images of the CUR@PA‐N‐2‐HACC‐Cys NPs; (c) pH stability of the CUR@PA‐N‐2‐HACC‐Cys NPs; (d) cumulative release in simulated gastric fluid (SGF) and simulated intestinal fluid (SIF). CUR, curcumin; Cys, cysteine; NPs, nanoparticles; SEM, scanning electron microscopy; TEM, transmission electron microscopy.

Both the ^1^H‐NMR (Figure S1a) and FT‐IR (Figure S1b) showed that the PA‐N‐2‐HACC‐Cys was successfully synthesized.^44^, ^45^, ^46^, ^47^, ^48^, ^49^ For the PA‐N‐2‐HACC‐Cys copolymer, PA forms a hydrophobic core to encapsulate low‐solubility CUR, which is subsequently stabilized by a hydrophilic N‐2‐HACC shell. The grafted Cys can form intermolecular or intramolecular disulfide bonds. When the dispersion medium pH was 4.5, the average particle size, polydispersity index (PDI), and zeta potentials of the CUR@PA‐N‐2‐HACC‐Cys NPs were 213.2 ± 4.78 nm, 0.20 ± 0.01, repectively (Figure 1a), implying that the introduction of hydrophobic PA into the polymer chains formed a hydrophobic core; and the positively charged hydrophilic N‐2‐HACC turned toward the external aqueous phase, making the whole NPs exhibiting a positively charged characteristics. By comparing different NPs (Table S1), we found that the particle size of the NPs was larger due to CUR encapsulation; and the particle size of PA‐N‐2‐HACC‐Cys NPs was smaller than that of PA‐N‐2‐HACC NPs, which might be due to the tighter internal structure of NPs caused by the introduction of Cys. Scanning electron microscopy (SEM) and transmission electron microscopy (TEM) images showed that the CUR@PA‐N‐2‐HACC‐Cys NPs were spherical, uniform in size, and well dispersed (Figure 1b). Moreover, according to high‐performance liquid chromatography (HPLC) results, the average peak area was linearly regressed with CUR concentration, and the regression equation was obtained (*y* = 0.6678*x* + 0.2871) (*R* ^2^ = 0.9999). The encapsulation efficiency (EE) and loading capacity (LC) were calculated as 85.10 ± 1.43% and 9.41 ± 0.73% respectively by regression equation, indicating that CUR was efficiently encapsulated into the PA‐N‐2‐HACC‐Cys NPs. In addition, to investigate whether the prepared FITC‐labeled polymer NPs can be used for in vitro and in vivo tracking, the in vitro leakage of FITC‐labeled NPs was measured. As shown in Figure S2, the leakage was very minimal and slow, which can be used for NPs tracking in vitro.

### 2.2 Stability of the CUR@PA‐N‐2‐HACC‐Cys NPs

The stability of the NPs is a prerequisite for oral administration. We investigated the stability of the CUR@PA‐N‐2‐HACC‐Cys NPs in buffers with various pH (1.2, 4.5, 6.0, 6.8, and 7.4), and these pH‐varied buffers were used to simulate different environments corresponding to gastric fluid (pH 1.2), lysosomes (pH 4.5), early endosomes (pH 6.0), intestinal fluid (pH 6.8), and blood plasma (pH 7.4). Figure 1c shows that almost no change in the particle size of CUR@PA‐N‐2‐HACC‐Cys NPs was observed in different simulated biological fluids for 12 h, confirming pH stability of the CUR@PA‐N‐2‐HACC‐Cys NPs. These results indicated that the CUR@PA‐N‐2‐HACC‐Cys NPs might be a promising delivery system, which could retain the stability of the loaded drugs and smoothly pass through the gastrointestinal tract. It is worth noting that the zeta potential of the NPs changed from positive to negative as the pH of buffers increased from 6.0 to 6.8, suggesting that as the NPs move from the upper to the lower digestive tract, the change in surface charge of NPs could help them penetrate the mucus quickly, thus preventing the strong adhesion from removing the embedded NPs with the renewal of mucus.^10^

### 2.3 In vitro release of the CUR@PA‐N‐2‐HACC‐Cys NPs

After oral administration, the drug must pass through the gastrointestinal tract before it can be effectively absorbed. Therefore, the drug release of the CUR@PA‐N‐2‐HACC‐Cys NPs was assessed by simulating digestive tract conditions in vitro. In our previous study, we have explored in vitro release of the CUR@PA‐N‐2‐HACC NPs and it was reported that the CUR@PA‐N‐2‐HACC NPs exhibited a slow‐release effect.^49^ In the present study, we also investigated in vitro release of the CUR@PA‐N‐2‐HACC‐Cys NPs (Figure 1d) and the results showed that the release rate of the CUR@PA‐N‐2‐HACC‐Cys NPs was slow, and the cumulative release amount was about 17% in SGF. The reason might be due to the presence of hydrophobic PA core, intermolecular hydrogen bonds, ionization of ammonium groups, and the formation of disulfide bonds in or outside the polymers.^49^ In addition, when the CUR@PA‐N‐2‐HACC‐Cys NPs were transferred to SIF, the release of CUR was triggered, and the CUR@PA‐N‐2‐HACC‐Cys NPs presented a sustained and controlled release behavior up to 24 h, increasing from 18% to almost 52% in the final of the experiment, which demonstrated a pH‐response behavior. The results suggested that CUR could be released from CUR@PA‐N‐2‐HACC‐Cys NPs in response to the intestinal environment. The pH‐response release manner could be explained since most of the ammonium groups in the N‐2‐HACC units were neutralized at intestinal pH, leading to the destruction of the hydrophilic shell of the nanomicelles, which might allow the CUR release.^49^ Therefore, the sustained and controlled release of the CUR@PA‐N‐2‐HACC‐Cys NPs could contribute to the absorption of CUR in the intestine, thus improving its bioavailability.

The release kinetics model showed that the CUR@PA‐N‐2‐HACC‐Cys NPs could release the CUR according to the diffusion mechanism in both SGF and SIF release medium (Table 1). By comparing the correlation coefficients (*r* ^2^) obtained from each release kinetics model, we concluded that the first‐order model was highly correlated with CUR release in SGF. However, the Korsmeyer–Peppas model was highly correlated with CUR release in SIF, referring to highest values of correlation coefficients. In addition, the major release mechanisms of the CUR@PA‐N‐2‐HACC‐Cys NPs in SGF and SIF was Super Case II transport and non‐Fickian transport, respectively, according to the value of “*n*” diffusional exponent.

**TABLE 1:** Release constants (*n*) and correlation coefficient values (*r* ^2^) obtained by fitting curcumin release data to zero‐order, first‐order, Higuchi, and Korsmeyer–Peppas.

Medium	Zero‐order	First‐order	Higuchi	Korsmeyer–Peppas		Release mechanism
	*r* ^2^	*r* ^2^	*r* ^2^	*r* ^2^	*n*	
SGF	0.884	0.996	0.820	0.965	1.667	Super Case II transport
SIF	0.915	0.883	0.947	0.956	0.704	Non‐Fickian diffusion

Abbreviations: SGF, simulated gastric fluid; SIF, simulated intestinal fluid.

### 2.4 Interaction between the PA‐N‐2‐HACC‐Cys NPs and mucus

**FIGURE 2:**
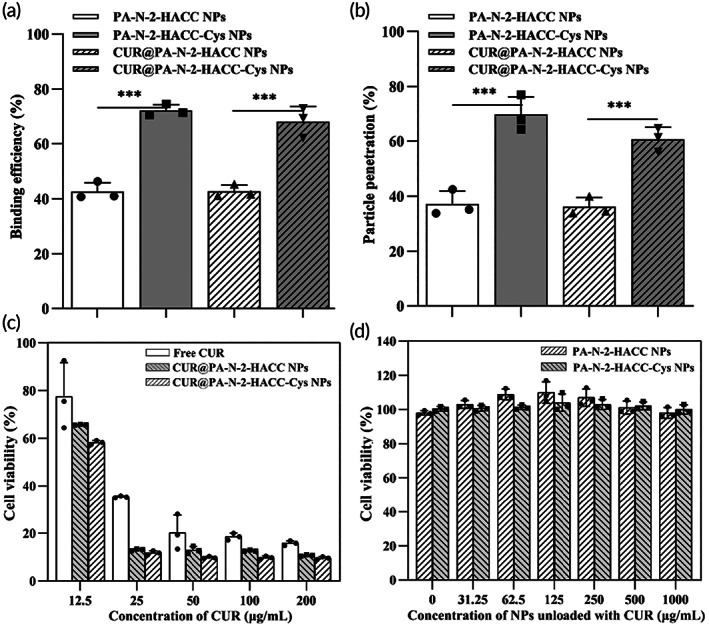
(a, b) Mucin‐binding efficiency and penetration percentage of the NPs loaded or unloaded with CUR; (c) in vitro cytotoxicity of the PA‐N‐2‐HACC‐Cys NPs and PA‐N‐2‐HACC NPs loaded with CUR against HepG‐2 at the different concentrations after 24 h of incubation; (d) cell viability of HT29‐MTX after incubation with the different blank NPs. All the experiments were performed in triplicate. CUR, curcumin; Cys, cysteine; NPs, nanoparticles.

For effective absorption into the blood capillary of intestinal villi, orally administrated NPs loaded with drugs need to overcome the mucus barrier that covers the intestinal epithelium.^50^ Therefore, we investigated the interaction between the NPs loaded or unloaded with CUR and mucus by mucus‐binding and mucus‐penetrating assay. Figure 2a demonstrates that the binding efficiency of the PA‐N‐2‐HACC NPs, PA‐N‐2‐HACC‐Cys NPs, CUR@PA‐N‐2‐HACC NPs and CUR@PA‐N‐2‐HACC‐Cys NPs was 42.7 ± 3.0%, 72.2 ± 2.0%, 40.7 ± 2.3% and 68.1 ± 5.4%, respectively. The difference in binding efficiency indicated that the Cys‐modified PA‐N‐2‐HACC‐Cys NPs had more vital interaction with the mucus fibers.^37^ Except for the electrostatic interaction, the formation of disulfide bonds between thiol groups of Cys and the glycoproteins of the mucus layer also played a role in mucoadhesion, which was much stronger than the electrostatic interaction between the N‐2‐HACC and mucus layer.^51^ These results indicated that the PA‐N‐2‐HACC‐Cys NPs could prolong the residence time of CUR in the mucus‐rich intestine by adhering to the mucus layer, thus achieving drug mucosal delivery.

Mucus‐penetrating ability of the NPs loaded or unloaded with CUR was assessed using the transwell system. The particle size of NPs is critical to allow particles to cross the mucus, avoid rapid clearance, and ultimately enhance transmucosal transport.^14^ Figure 2b shows that the percentage of particle penetration for the PA‐N‐2‐HACC NPs, PA‐N‐2‐HACC‐Cys NPs, CUR@PA‐N‐2‐HACC NPs, and CUR@PA‐N‐2‐HACC‐Cys NPs was 37.2 ± 0.7%, 69.7 ± 6.6%, 35.9 ± 3.3%, and 61.4 ± 4.3% at 4 h, respectively. Compared with the CUR@PA‐N‐2‐HACC NPs, the CUR@PA‐N‐2‐HACC‐Cys NPs of smaller particle size could penetrate the mucus layer more quickly and efficiently (Table S1). Moreover, the enhanced permeation effect of PA‐N‐2‐HACC‐Cys NPs could be explained by the immobilization of thiol groups;^52^ and the introduction of Cys not only enhanced the interaction between NPs and mucin but also formed disulfide bonds with mucin and attenuated the interaction between mucin, thus “diluting” mucus and enhancing the penetration ability of NPs.^53^

### 2.5 Cell viability assay

Figure 2c reveals that all the formulations showed significant cytotoxicity against HepG‐2 after incubation for 24 h. Cell viability of HepG‐2 after incubation with the free CUR, CUR@PA‐N‐2‐HACC, and CUR@PA‐N‐2‐HACC‐Cys NPs was 77.5%, 65.7%, and 57.3%, respectively, when the CUR concentration was 12.5 μg/mL. As the CUR concentration was increased, the CUR@PA‐N‐2‐HACC‐Cys NPs and CUR@PA‐N‐2‐HACC NPs exhibited higher cytotoxicity as compared with the free CUR. This finding might be attributed to the introduction of a positive charge of −N^+^(CH_3_)_3_ and Cys in the N‐2‐HACC, which allowed it to bind to negatively charged cell membranes, resulting in more significant toxicity to cancer cells.^54^ In addition, as shown in Figure 2d, the cell viability of HT29‐MTX was above 80%. The cytotoxicity of the blank PA‐N‐2‐HACC‐Cys NPs unloaded with CUR was negligible as the testing concentration ranged from 31.25 to 1000 μg/mL, indicating that the PA‐N‐2‐HACC‐Cys NPs had excellent biocompatibility and potential as an effective platform for oral delivery.

### 2.6 Cellular uptake of the PA‐N‐2‐HACC‐Cys NPs

**FIGURE 3:**
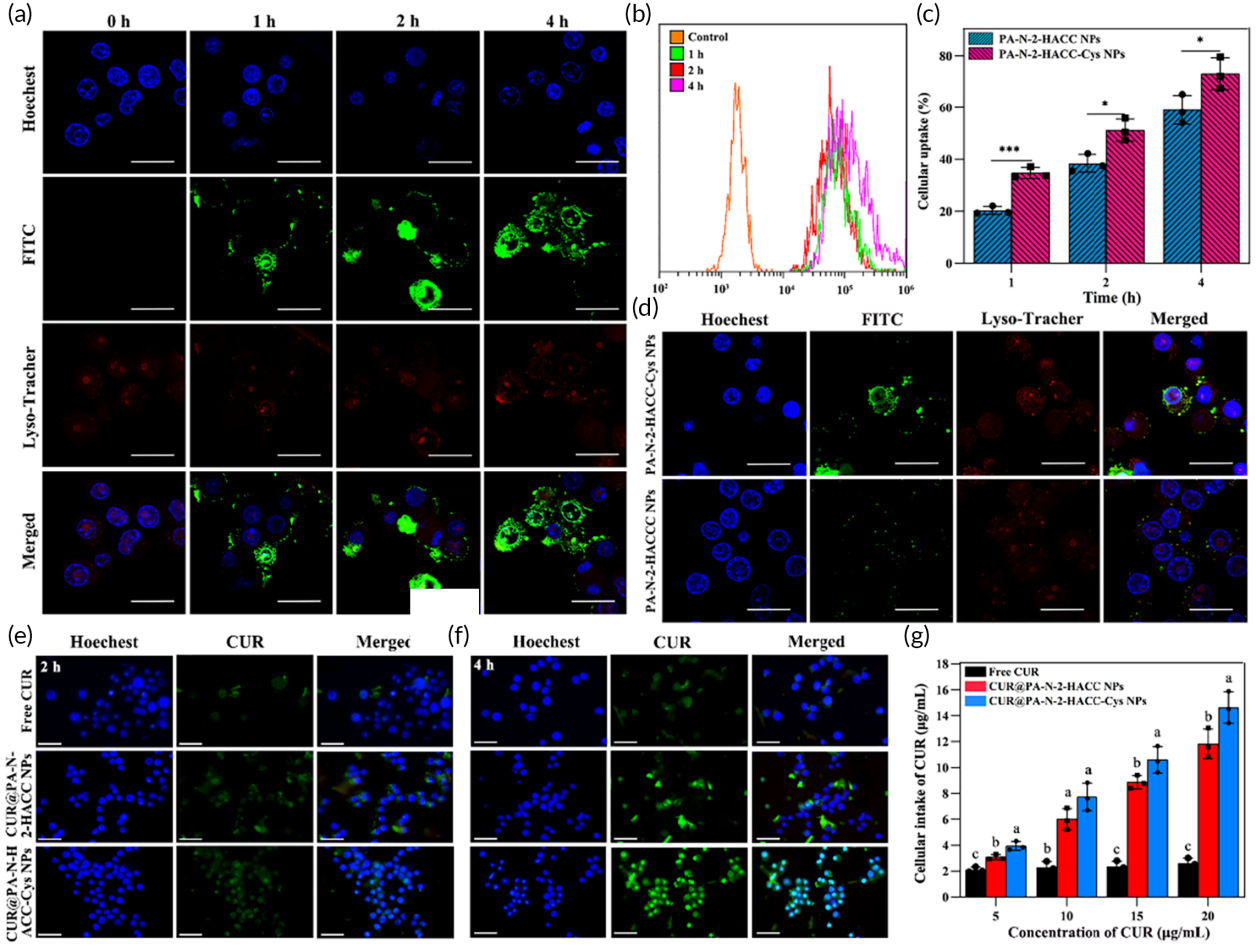
Qualitative and quantitative analysis of cellular uptake of the NPs loaded or unloaded with CUR in HT29‐MTX (*n* = 3). (a) CLSM images indicating the time‐dependent cellular uptake of the PA‐N‐2‐HACC‐Cys NPs; (b) FCM curves of HT29‐MTX treated with the PA‐N‐2‐HACC‐Cys NPs for 0, 1, 2, and 4 h, respectively; (c) percentage of cells containing FITC‐NPs after treatment with the PA‐N‐2‐HACC and PA‐N‐2‐HACC‐Cys NPs; (d) fluorescence images of cell internalization profiles of the PA‐N‐2‐HACC, PA‐N‐2‐HACC‐Cys NPs for 2 h (scale bar = 20 μm); (e, f) intracellular uptake of CUR from the free CUR, CUR@PA‐N‐2‐HACC NPs or CUR@PA‐N‐2‐HACC‐Cys NPs for 2 and 4 h (scale bar = 20 μm); (g) cellular uptake of the free CUR and different preparations at 4 h. CUR, curcumin; Cys, cysteine; NPs, nanoparticles.

The epithelium is another significant absorption barrier for the oral administration of NPs. Therefore, we used HT29‐MTX to study the epithelium uptake of the NPs. The fluorescence signal in HT29‐MTX was significantly increased with prolonged incubation time (Figure 3a) at the same dose of fluoresceine isothiocyanate (FITC) labeled PA‐N‐2‐HACC‐Cys NPs. Flow cytometry (FCM) (BD, BD FACSVERSE, USA) further confirmed effective cellular uptake of FITC‐labeled PA‐N‐2‐HACC‐Cys NPs by HT29‐MTX in a time‐dependent manner (Figure 3b), which was consistent with the observation by confocal laser scanning microscope (CLSM). In addition, staining of late endosomes and lysosomes by LysoTracker (Red fluorescence) revealed endolysosomal transport of internalized FITC‐labeled PA‐N‐2‐HACC‐Cys NPs in HT29‐MTX because co‐localization of green and red fluorescence signals was observed after cells were treated with NPs for 1 or 2 h. In particular, we observed that green fluorescence signals entered the nucleus, and most of the green and red fluorescence signals were not co‐located when the cells were incubated with the PA‐N‐2‐HACC‐Cys NPs for 4 h (Figure 3a), indicating that the Cys‐modified PA‐N‐2‐HACC‐Cys NPs could realize the function of lysosomal escape. We also found that the PA‐N‐2‐HACC‐Cys NPs showed higher cellular uptake efficiency compared with the PA‐N‐2‐HACC NPs (Figure 3c,d). The internalization efficiency of the PA‐N‐2‐HACC‐Cys NPs at each test time point was 1.2, 1.3, and 1.7 times higher compared with the PA‐N‐2‐HACC NPs, respectively (Figure 3c). It was worth noting that the fluorescence signals of HT29‐MTX cells treated with the PA‐N‐2‐HACC‐Cys NPs were also significantly stronger compared with those treated with the PA‐N‐2‐HACC NPs (Figure 3d). The above results indicated that the PA‐N‐2‐HACC‐Cys NPs could effectively promote epithelial uptake. To sum up, the PA‐N‐2‐HACC‐Cys NPs could enhance cellular uptake via endolysosomal transport.

Moreover, to evaluate the internalization mechanism of the free CUR, CUR@PA‐N‐2‐HACC NPs and CUR@PA‐N‐2‐HACC‐Cys NPs, the cellular uptake of the NPs in HT29‐MTX was observed by CLSM. As shown in Figure 3e, HT9‐MTX treated with the CUR@PA‐N‐2‐HACC NPs and CUR@PA‐N‐2‐HACC‐Cys NPs exhibited gradual increase in fluorescence intensity from 2 to 4 h. Notably, the fluorescence intensity of CUR@PA‐N‐2‐HACC‐Cys NPs was observed to be stronger than that of the CUR@PA‐N‐2‐HACC NPs. On the other hand, HT9‐MTX treated with the free CUR exhibited a weak fluorescence intensity over time, indicating the PA‐N‐2‐HACC NPs and PA‐N‐2‐HACC‐Cys NPs resulted in a more extensive and prolonged exposure of CUR to HT9‐MTX relative to the free CUR. In addition, Figure 3g shows that the cellular intake of CUR in the CUR@PA‐N‐2‐HACC‐Cys NPs and CUR@PA‐N‐2‐HACC NPs was higher than that in the free CUR group within the range of experimental concentration, and compared with the CUR@PA‐N‐2‐HACC NPs, the CUR@PA‐N‐2‐HACC‐Cys NPs had a higher cellular uptake (Figure S3). The results might be attributed to the formation of inter or intramolecular disulfide bonds by Cys, further increasing the interaction with cell membrane to enhance cellular uptake.^37^, ^38^

### 2.7 Transepithelial transport of the NPs loaded or unloaded with CUR

**FIGURE 4:**
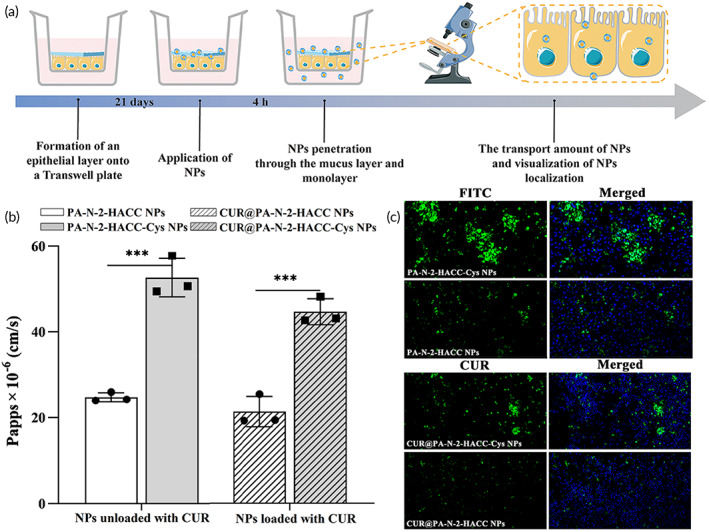
Transepithelial transport of PA‐N‐2‐HACC NPs and PA‐N‐2‐HACC‐Cys nanoparticles (NPs) on HT29‐MTX monolayer (*n* = 3). (a) Schematic diagram of transepithelial transport of NPs; (b) *P*_app_ value of NPs transepithelial transport; (c) 2D fluorescence images in the middle of an HT29‐MTX monolayer after incubation with NPs for 4 h. Scale bar = 200 μm.

To assess the ability of the NPs loaded or unloaded with CUR to overcome mucus and epithelial barriers, we explored the transepithelial transport of NPs loaded or unloaded with CUR using a transwell plate (Figure 4a). HT29‐MTX is a type of mucus‐secreting cell, and its monolayer constructed on transwell support is commonly used as an epithelial barrier model for mucous secretion.^55^ Therefore, the transported amount of the FITC‐labeled PA‐N‐2‐HACC NPs and FITC‐labeled PA‐N‐2‐HACC‐Cys NPs on the HT29‐MTX monolayer was measured by fluorescence spectrophotometer and expressed as apparent permeability coefficient (*P*_app_). Figure 4b shows that the PA‐N‐2‐HACC NPs owned the lower *P*_app_ value of 24.76 × 10^−6^ cm/s, while the *P*_app_ value of PA‐N‐2‐HACC‐Cys NPs was 52.6 × 10^−6^ cm/s, which was 2.12 times higher compared with PA‐N‐2‐HACC NPs. In addition, we measured the transepithelial transport of CUR@PA‐N‐2‐HACC‐Cys NPs, *P*_app_ value of the CUR@PA‐N‐2‐HACC‐Cys NPs was 2.12 times higher compared with the CUR@PA‐N‐2‐HACC NPs. Overall, Cys‐modified delivery carrier seemed to be beneficial for delivering CUR.

In addition, a transwell insert containing the HT29‐MTX monolayer was placed on a slide and visualized with CLSM (Figure 4c). HT29‐MTX monolayer treated with the FITC‐labeled PA‐N‐2‐HACC‐Cys NPs exhibited more vigorous green fluorescence intensity, demonstrating the excellent ability of PA‐N‐2‐HACC‐Cys NPs to effectively cross the mucus and epithelial barrier. In contrast, few FITC‐labeled PA‐N‐2‐HACC NPs were observed on the HT29‐MTX monolayer due to their poor permeability. The same results were observed for HT29‐MTX monolayer treated with the CUR@PA‐N‐2‐HACC‐Cys NPs and CUR@PA‐N‐2‐HACC NPs. More importantly, SEM image also indicated that the CUR@PA‐N‐2‐HACC‐Cys NPs showed more complete morphology in the lower chamber after incubation with HT29‐MTX (Figure S4). These results confirmed that the transepithelial transport efficiency of CUR@PA‐N‐2‐HACC‐Cys NPs was much stronger than that of the CUR@PA‐N‐2‐HACC NPs. We proposed that when the CUR@PA‐N‐2‐HACC‐Cys NPs approached cells, the NPs could penetrate the mucus layer and continuously transport through the epithelial cell layer due to their interaction with proteins containing disulfide bonds.

### 2.8 In vivo absorption

**FIGURE 5:**
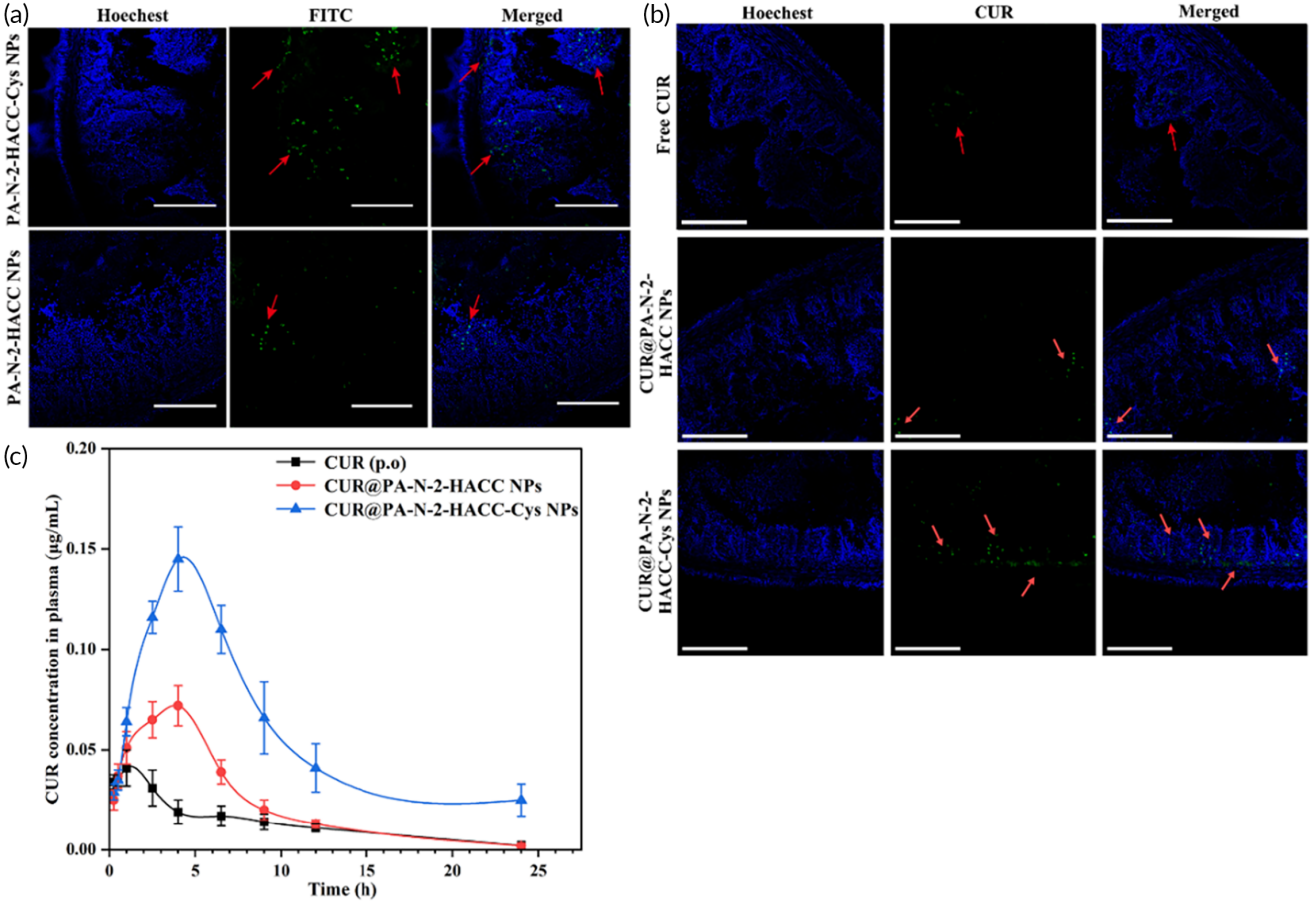
(a) Fluorescence images of intestinal tissue after administration of the FITC‐labeled PA‐N‐2‐HACC NPs and PA‐N‐2‐HACC‐Cys NPs for 2 h (*n* = 3). Scale bars: 200 μm; (b) Fluorescence images of intestinal tissue after administration of the free CUR, CUR@PA‐N‐2‐HACC NPs and CUR@PA‐N‐2‐HACC‐Cys NPs for 2 h (*n* = 3). Scale bars: 200 μm; (c) Plasma CUR concentration‐time curve of free CUR, CUR@PA‐N‐2‐HACC NPs, and CUR@PA‐N‐2‐HACC‐Cys NPs at a CUR dose of 50 mg/kg (*n* = 3). CUR, curcumin; Cys, cysteine; NPs, nanoparticles.

To further evaluate the absorption behavior of the PA‐N‐2‐HACC NPs and PA‐N‐2‐HACC‐Cys NPs, we performed in vivo absorption of FITC‐labeled PA‐N‐2‐HACC NPs and FITC‐labeled PA‐N‐2‐HACC‐Cys NPs into epithelium on SD rats, and the small intestinal tissue treated with the NPs was visualized using CLSM. Figure 5a shows that as compared with the PA‐N‐2‐HACC NPs, the PA‐N‐2‐HACC‐Cys NPs displayed a higher green fluorescence signal in the interior of intestinal villi (red arrows), indicating that the PA‐N‐2‐HACC‐Cys NPs have the potential to pass across the epithelium into the blood circulation, which was attributed to the improved mucoadhesion caused by the formation of disulfide bonds between Cys and mucins.^37^ Moreover, Cys not only gave them a high affinity for intestinal absorption but also conferred mucus permeability to PA‐N‐2‐HCACC‐Cys NPs.^56^, ^57^ Notably, in vivo absorption study of the CUR@PA‐N‐2‐HACC NPs and CUR@PA‐N‐2‐HACC‐Cys NPs has obtained consistent result. As shown in Figure 5b, the free CUR group presented weak green fluorescence signal, and the CUR@PA‐N‐2‐HACC‐Cys NPs showed extensive green fluorescence signal in both mucosa and submucosa, as compared with the CUR@PA‐N‐2‐HACC NPs and free CUR. Taken together, mucoadhesion, permeability, and transepithelial transport of PA‐N‐2‐HACC‐Cys NPs were found, thus demonstrating its potential for oral delivery.

### 2.9 Pharmacokinetic study

We investigated the pharmacokinetics of the free CUR, CUR@PA‐N‐2‐HACC NPs, and CUR@PA‐N‐2‐HACC‐Cys NPs via oral administration to the SD rats (Figure 5b). For years, the unique pharmacokinetic characteristics and the mode of action of CUR remain to be of great interest. One study has been reported that only slight changes were observed in peripheral blood of patients after taking 8000 mg of curcumin daily.^58^ Similarly, in another study, the concentration of CUR in peripheral circulation was at nmol/L level after receiving 3600 mg of CUR daily in patients with metastatic colon cancer.^59^ In the study, the CUR@PA‐N‐2‐HACC‐Cys NPs significantly improved the oral bioavailability of CUR. The maximum drug concentration (*C*_max_) of the CUR@PA‐N‐2‐HACC‐Cys NPs group was 0.145 ± 0.02 μg/mL, and it was the highest among all formulations (Table 2). The area under curve (AUC) value of the CUR@PA‐N‐2‐HACC‐Cys NPs group was 6.42‐fold and 2.45‐fold higher compared with the free CUR and CUR@PA‐N‐2‐HACC NPs groups, respectively, indicating that the highest absorption of CUR was in the PA‐N‐2‐HACC‐Cys NPs group. Time to peak (*T*_max_) of the free CUR, CUR@PA‐N‐2‐HACC NPs, and CUR@PA‐N‐2‐HACC‐Cys NPs was 1, 4, and 4 h, respectively, indicating that nano‐carrier encapsulation of CUR had mucoadhesive properties, which caused the CUR to be released with a considerable lag‐time or delay.^60^ Half‐life (*T*_1/2_) of the CUR@PA‐N‐2‐HACC‐Cys NPs group was 7.34 ± 0.30 h, which was significantly prolonged compared with the free CUR (3.27 ± 0.32 h) and CUR@PA‐N‐2‐HACC NPs groups (6.25 ± 0.27 h) after oral administration. Additionally, the CUR@PA‐N‐2‐HACC‐Cys NPs presented the longest mean residence time (MRT) and lowest clearance (CL), which could significantly maintain a particular blood concentration for a long time.^60^ In conclusion, the pharmacokinetic parameters of CUR after oral administration in SD rats showed that administration in the form of nano‐systems improved oral bioavailability of the CUR compared with the free CUR. The advantages of the CUR@PA‐N‐2‐HACC‐Cys NPs could be attributed to the synergy effect of positively charged quaternary ammonium salt, thiol‐mediated mucoadhesion, and the excellent permeability by thiol groups.^36^ After oral administration, the CUR@PA‐N‐2‐HACC‐Cys NPs firstly prevented CUR from degrading in the gastrointestinal tract and subsequently passed smoothly through the upper digestive tract into the small intestine; the mucoadhesion of CUR@PA‐N‐2‐HACC‐Cys NPs prolonged the intestinal retention time; the CUR@PA‐N‐2‐HACC‐Cys NPs with thiol groups quickly penetrated mucus into the mucosa; and the cationic N‐2‐HACC opened the TJs between intestinal epithelia for transepithelial transport, thus improving the oral bioavailability of CUR.

**TABLE 2:** Pharmacokinetic parameters of the free CUR, CUR@PA‐N‐2‐HACC NPs, and CUR@PA‐N‐2‐HACC‐Cys NPs after oral administration in SD rats (CUR dose of 50 mg/kg, mean ± SD, *n* = 3).

Parameter	CUR (p.o)	CUR@PA‐N‐2‐HACC NPs	CUR@PA‐N‐2‐HACC‐Cys NPs
AUC_0‐∞_ (μg × h/mL)	0.252 ± 0.11	0.66 ± 0.12**	1.62 ± 0.21***
*C*_max_ (μg/mL)	0.041 ± 0.01	0.072 ± 0.01	0.145 ± 0.02***
*T*_max_ (h)	1	4	4
*t*_1/2_ (h)	3.27 ± 0.32	6.25 ± 0.27***	7.34 ± 0.30***
CL (L/h/kg)	46.96 ± 1.12	27.22 ± 0.63***	11.10 ± 0.22***
MRT (h)	4.71 ± 0.48	9.02 ± 0.79***	10.59 ± 0.83***

*Note*: **p < 0.01; ***p < 0.001 relative to CUR (p.o) group.

Abbreviations: CUR, curcumin; Cys, cysteine; NPs, nanoparticles.

### 2.10 In vivo therapeutic effect of the CUR@PA‐N‐2‐HACC‐Cys NPs against colitis

**FIGURE 6:**
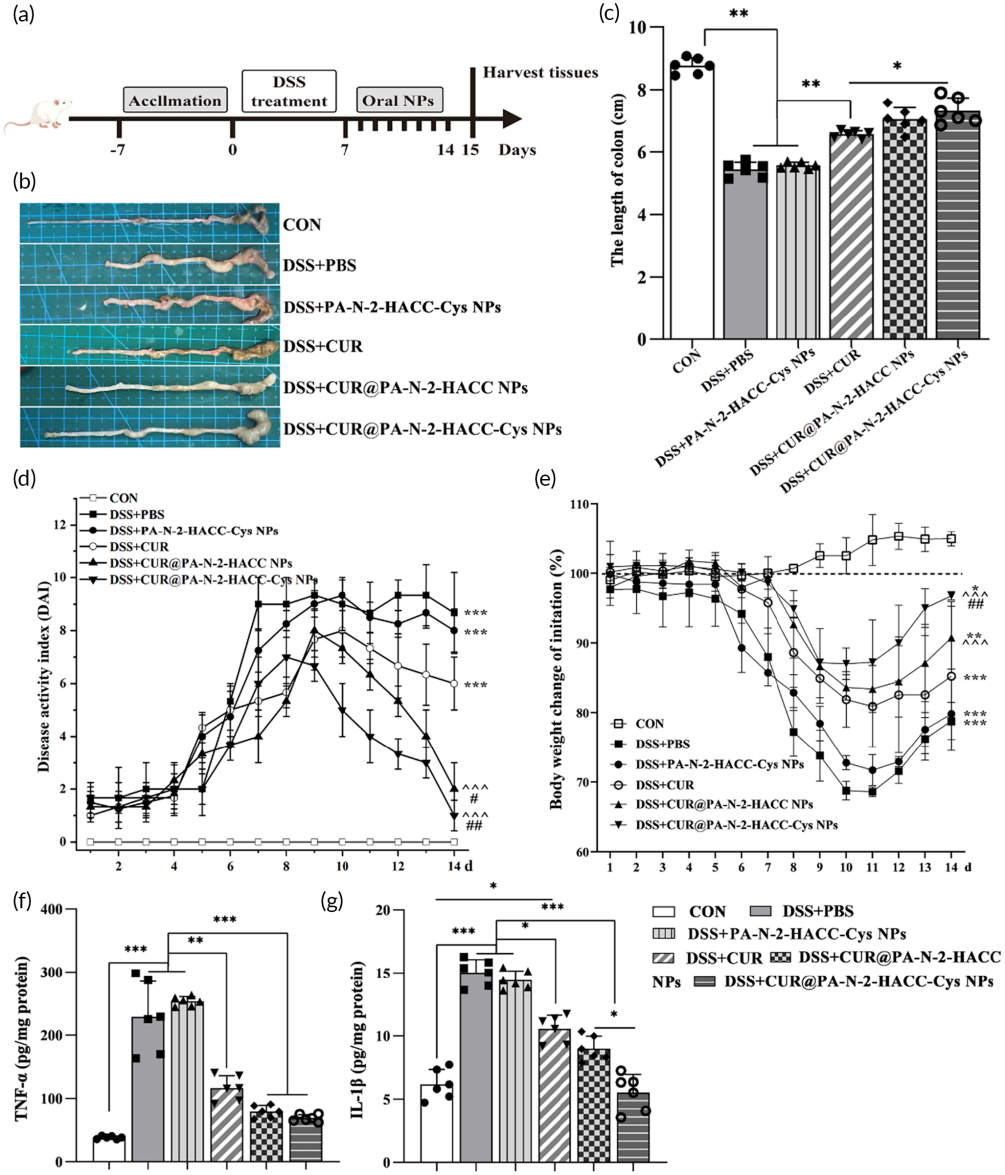
In vivo therapeutic effect of the CUR@PA‐N‐2‐HACC‐Cys NPs against colitis (*n* = 8). (a) Diagram illustrating the mouse model of colitis treated in this study; (b) photographs of colons; (c) the lengths of colons; (d) kinetics of daily DAI scores; (e) daily variations of mice body weight (*p < 0.05, **p < 0.01, ***p < 0.001 relative to CON group; ^*p* < 0.05, ^^*p* < 0.01, ^^^*p* < 0.001 relative to DSS + PBS group; ^#^ *p* < 0.05, ^*##*^ *p* < 0.01 relative to DSS + CUR group); (f, g) Expression of TNF‐α and IL‐1β in colonic tissue of colitis mice. CUR, curcumin; Cys, cysteine; DAI, disease activity index; NPs, nanoparticles.

We further evaluated the efficacy of the CUR@PA‐N‐2‐HACC‐Cys NPs on DSS‐induced colitis in vivo (Figure 6a). Compared with the CON group, mice in the DSS + PBS group presented severe clinical symptoms, such as hematochezia and diarrhea, accompanied by significantly shortened colon length (Figure 6b,c), elevated disease activity index (DAI, the detailed evaluation criteria were shown in Table 3) (Figure 6d), and body weight loss (Figure 6e). Different CUR formulation interventions alleviated the above symptoms to some extent, especially the CUR@PA‐N‐2‐HACC‐Cys NPs.

**TABLE 3:** Colitis disease activity index evaluation criteria.

Score	Character of stool	The degree of hematochezia	Weight loss
0	Normal	Stool color is normal	No weight loss
1	Soft stool	Brown stool	Weight loss 1%–5%
2	Unformed stool	Light red stool	Weight loss 5%–10%
3	Watery stool	Deep red stool	Weight loss 10%–15%
4			Weight loss >15%

Furthermore, the secreted levels of the main pro‐inflammatory cytokines (TNF‐α and IL‐1β) in colonic tissues were noticeably increased in the DSS + PBS group, compared with that in the normal group (Figure 6f,g). The secreted levels of inflammatory cytokines in different CUR formulation interventions were lower than those in the DSS + PBS group, whereas the DSS + CUR@PA‐N‐2‐HACC‐Cys NPs groups showed the most pronounced improvement. Compared with the CUR@PA‐N‐2‐HACC NPs, the CUR@PA‐N‐2‐HACC‐Cys NPs significantly decreased the concentration of IL‐1β in the colon of mice. However, oral Blank PA‐N‐2‐HACC NPs failed to decrease the levels of pro‐inflammatory cytokines in the colon of DSS‐treated mice.

**FIGURE 7:**
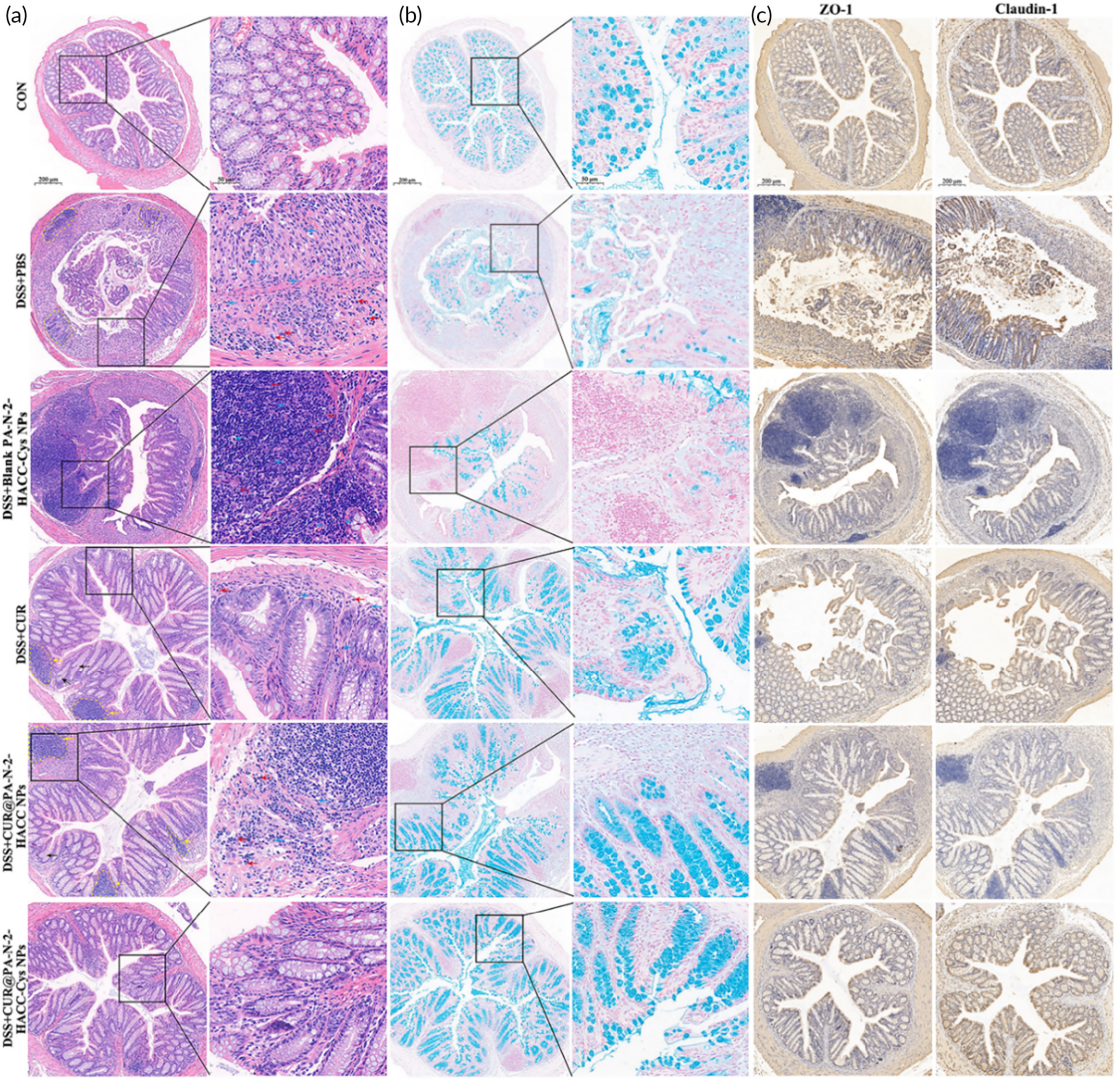
**(**a) Representative images of H&E stained colon sections (blue arrowhead, basal neutrophils; red arrowhead, basal plasmacytosis; yellow arrowhead, basal lymphoid aggregates; black arrowhead, branching of crypts); (b) representative images of Alcian blue‐stained inner mucus layer of colonic sections; (c) representative photographs of IHC staining for ZO‐1 and Claudin‐1.

Histological analysis of the excised colons from the DSS + PBS group in comparison with those from the CON group further revealed clear signs of inflammation, including abundant inflammatory cells infiltrated into colonic submucosa, vast crypt disappearance, depletion of goblet cells, disruption of mucosal structures, and ulcer formation (Figure 7a). These findings indicated that the colitis mice model was successfully established. Clinical symptoms, such as body weight loss, colon length shortening, and DAI increase, were significantly improved in the DSS + CUR, DSS + CUR@PA‐N‐2‐HACC NPs, and DSS + CUR@PA‐N‐2‐HACC‐Cys NPs groups, but no significant remission of colitis was observed in the DSS + PA‐N‐2‐HACC‐Cys NPs group. Among them, the colonic mucosa morphology of mice in the DSS + CUR@PA‐N‐2‐HACC‐Cys NPs group was basically the same as that of healthy mice, the surface mucosa epithelium was basically recovered, goblet cells were increased, and inflammatory cell infiltration into mucosa and submucosa was significantly reduced. In addition, the clinical symptoms of mice in the DSS + CUR and DSS + CUR@PA‐N‐2‐HACC NPs groups were also improved to a certain extent. The colon mucosal hyperemia, edema, and intestinal wall thickening were alleviated, while inflammatory cell infiltration, distortion of crypts, and basal lymphocyte aggregation still existed.

Moreover, to assess the effect of CUR@PA‐N‐2‐HACC‐Cys NPs on the colonic mucosal barrier, mucin‐secreting goblet cells were measured using Alcian blue staining. DSS significantly reduced the thickness of colonic epithelial mucosa, and oral administration of the free CUR, CUR@PA‐N‐2‐HACC NPs, and CUR@PA‐N‐2‐HACC‐Cys NPs relieved the damage caused by DSS, among which the CUR@PA‐N‐2‐HACC‐Cys NPs group had the best therapeutic effect (Figure 7b). In addition, intestinal permeability is also regulated by the TJs of the intestine. As shown in Figure 7c, significant decline of the epithelial tight junction proteins Claudin‐1 and ZO‐1 was found in the DSS + PBS and DSS + PA‐N‐2‐HACC‐Cys NPs groups compared with that in the normal group, and different CUR formulation intervention had corresponding recovery, in which the colonic mucosa in the CUR@PA‐N‐2‐HACC‐Cys NPs group completely returned to normal levels. Overall, the CUR@PA‐N‐2‐HACC‐Cys NPs were superior to other treatment groups in the therapeutic efficacy against DSS‐induced colitis. These results indicated that the CUR@PA‐N‐2‐HACC‐Cys NPs had the potential to treat colitis.

## 3 MATERIALS AND METHODS

### 3.1 Materials

Curcumin, palmitic acid, cysteine, 1‐Ethyl‐3 (3‐dimethylaminopropyl) carbodiimide (EDC), *N*‐hydroxysuccinimide (NHS), and 4‐(dimethylamino)‐pyridin (DMAP) were purchased from Aladdin (Shanghai, China). Mucin type II from porcine stomach was purchased from Sigma (St. Louis, USA). Dextran sulfate sodium salt (DSS) (molecular mass 36–40 K) was purchased from MP Biologicals (Solon, USA).

### 3.2 Ethics statement

Twelve male SD rats (6–8 weeks, 230–250 g) and 40 male BALB/c mice (6–8 weeks, 20–25 g) were purchased from the Zhejiang Vital River Laboratory Animal Technology Co., Ltd. (SCXK (Z) 2019‐0001). All the animal studies were approved by the Institutional Animal Care and Use Committee of Taizhou University, Zhejiang, China (SYXK (Z) 2021‐0013). Care of laboratory animals and all animal experiments were in accordance with the “National Research Council's Guide for the Care and Use of Laboratory Animals.” Before the experiment, the animals were fasted for 24 h but allowed free access to water. The sex of animals has no effect on the susceptibility to DSS‐induced colitis, but male animal was more severely affected in the colon than female,^61^ Hence, we have selected male mice to establish colitis models.

### 3.3 Synthesis of the PA‐N‐2‐HACC‐Cys

**FIGURE 8:**
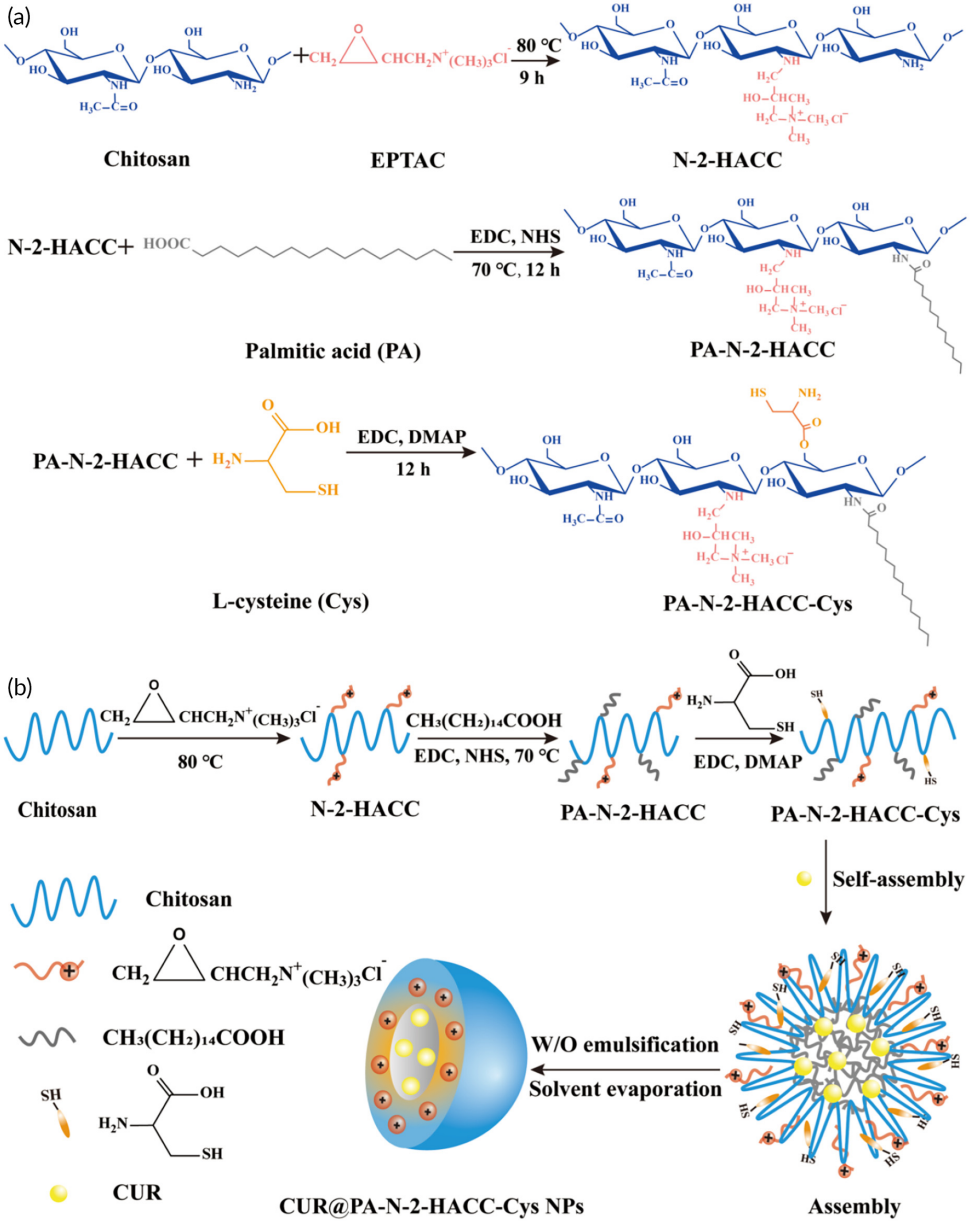
Schematic diagram of synthetic route of the PA‐N‐2‐HACC‐Cys and CUR@PA‐N‐2‐HACC‐Cys NPs. (a) Synthesis route of the PA‐N‐2‐HACC‐Cys; (b) formation process of the CUR@PA‐N‐2‐HACC‐Cys NPs by amphiphilic self‐assembly. CUR, curcumin; Cys, cysteine; NPs, nanoparticles.

The PA‐N‐2‐HACC was synthesized according to our previously published protocol.^49^ The PA‐N‐2‐HACC‐Cys was synthesized via esterification of hydroxyl groups on PA‐N‐2‐HACC and the carboxyl group of Cys (Figure 8a). Exactly 300 mg of the PA‐N‐2‐HACC and 400 mg of Cys were dispersed in 40 mL *N, N*‐Dimethylformamide, and then 800 mg of EDC and 20 mg of DMAP were added, followed by a reaction at room temperature for 24 h. Subsequently, the product was subjected to dialysis against deionized water (molecular weight cutoff of 7.5 K). The copolymer PA‐N‐2‐HACC‐Cys was lyophilized and stored at 4°C. The amount of PA and Cys bound to the polymer backbone, and characterization of the PA‐N‐2‐HACC‐Cys was compiled in the Supplementary Information.

### 3.4 Preparation of the PA‐N‐2‐HACC‐Cys NPs loaded‐CUR

The PA‐N‐2‐HACC‐Cys NPs loaded‐CUR (CUR@PA‐N‐2‐HACC‐Cys NPs) were prepared using the cosolvent evaporation method. Briefly, 7.5 mg of the PA‐N‐2‐HACC‐Cys was dissolved in 10 mL distilled water and 1 mL of the CUR in dichloromethane (1 mg/mL) was added dropwise into the above solution followed by ultrasonic at 90 w for 10 min (Sxsonic, FS‐1800N, Shanghai) and evaporation at 40°C under reduced pressure for 20 min (Ya Rong, RE52CS‐1, Shanghai). The final solution (pH 4.5) was centrifuged at 12,000 r/min for 30 min (Thermo Fisher, Sorvall ST 16R, German), and the residue was washed with ethanol and deionized water five times and then lyophilized. In order to investigate the effect of PA and Cys graft on PA‐N‐2‐HACC‐Cys NPs, CUR@PA‐N‐2‐HACC NPs were prepared as previously described. Figure 8b shows the amphiphilic self‐assembly process for forming the CUR@PA‐N‐2‐HACC‐Cys NPs. For in vitro and in vivo fluorescence imaging, FITC (F6120‐100 mg, Macklin) labeled NPs is described in the Supplementary Information.

#### 3.4.1 Particle size and morphology of the CUR@PA‐N‐2‐HACC‐Cys NPs

The size distribution, PDI, and zeta potential of the CUR@PA‐N‐2‐HACC‐Cys NPs were determined using dynamic light scattering (DLS) and Zeta potentiometer (Malvern instruments, ZEN3690//Nano ZS90, UK), respectively. The morphology of the CUR@PA‐N‐2‐HACC‐Cys NPs was observed by TEM (Thermo Fisher, Thermo Scientific Talos F200i, USA) and SEM (Hitachi, S‐4800, Japan).

#### 3.4.2 Measurement of encapsulation efficiency and loading capacity

The EE and LC were measured using HPLC according to previous reports.^62^, ^63^ Briefly, the CUR@PA‐N‐2‐HACC‐Cys NPs were centrifuged at 12,000 r/min for 30 min at 4°C. The supernatant was subjected to quantification by HPLC using a Kromasil‐C_18_ column (250 mm × 4.6 mm, 5 μm). The HPLC condition was set as follows: mobile phase of acetonitrile: water (containing 2% acetic acid) (52∶48, v/v); flow rate of 1.0 mL/min; detection wavelength of 426 nm. All the measurements were performed in triplicate. The EE and LC were calculated using Formulas (1) and (2), respectively.

(1) $$\mathrm{EE}\%=\frac{\text{Weight of}\;\mathrm{CUR}\;\text{added}-\text{Weight of free}\;\mathrm{CUR}}{\text{Weight of}\;\mathrm{CUR}\;\text{added}}\times 100.$$

(2) $$\mathrm{LC}\%=\frac{\text{Weight of}\;\mathrm{CUR}\;\text{added}-\text{Weight of free}\;\mathrm{CUR}}{\text{Total weight of}\;\mathrm{NPs}}\times 100.$$

### 3.5 Stability and in vitro release of the CUR@PA‐N‐2‐HACC‐Cys NPs

After incubation at different pH (1.2, 4.5, 6.0, 6.8, and 7.4) for 12 h, the NPs were measured to investigate stability by measuring the changes in particle size, PDI, and surface charge.^64^

#### 3.5.1 In vitro release

Exactly 2.0 g of NaCl and 3.2 g of pepsin were mixed with 7 mL of hydrochloric acid, and then the mixture was dissolved in 1000 mL of deionized water to obtain SGF (pH 1.2). SIF (pH 6.8) was prepared by dissolving 6.8 g of monopotassium phosphate and 10 g of pancreatin in 500 of deionized water, followed by pH adjustment to 6.8 with 0.1 mol/L sodium hydroxide solution, and deionized water was added to the solution to obtain 1000 mL of the SIF. The dialysis bag was filled with 4 mL of the CUR@PA‐N‐2‐HACC‐Cys NPs solution (1 mg/mL) and immersed in 40 mL of the SGF medium at 37°C. After 2 h, the dialysis bag was transferred from the SGF medium to 40 mL of the SIF medium, and the dialysis was continued for 24 h. Subsequently, 400 μL of the release medium was removed at different time points (0.5, 1, 2, 4, 8, 12, and 24 h) to measure the concentration of CUR. The dissolution medium was kept constant at 40 mL by adding a fresh dissolution medium. All the measurements were performed in triplicate. The release of CUR in the CUR@PA‐N‐2‐HACC NPs was calculated using Formula (3), where *V*_*e*_, *V*_0_, *M*, and *n* are the volume of each equal portion (400 μL), the total volume of the medium (40 mL), the quality of the CUR according to the conversion of LC using Formula (2), and the time of the measurement, respectively, while *C*_*i*_ or *C*_*n*_ represents the concentration of CUR in the release medium at the appointed time.^62^

(3) $$\mathrm{CUR}\;\text{release}\;\left(\%\right)=\frac{V_{e}\sum_{1}^{n-1}C_{i}+V_{0}C_{n}}{M}\times 100.$$

#### 3.5.2 Release kinetic model

To explain the release mechanism of CUR in the CUR@PA‐N‐2‐HACC NPs, the sustained release of CUR from the CUR@PA‐N‐2‐HACC NPs in SGF and SIF was fitted using four different types of kinetic models: zero‐order model, first‐order model, Korsmeyer–Peppas model,^65^ and Higuchi model^66^ according to the following Equations ((4), (5), (6), (7)). When *n* = 1, the zero‐order model is suitable. When *n* = 0.5, to the Higuchi model applies. *Q*, *K*_0_, *K*_1_, *K*_H_, and *K*_P_ are the cumulative percentage of released CUR at time t and rate constants of CUR release for zero‐order, first‐order, Higuchi, and Korsmeyer–Peppas models, respectively. Moreover, n gives an indication of the release mechanism. *n* = 0.45, 0.45 < *n* < 0.89, *n* = 0.89, and *n* > 0.89 suggest that the drug is released from NPs via Fickian diffusion, non‐Fickian diffusion, Case II transport, and Super Case II transport, respectively.

(4) $$Q=K_{0}t.$$

(5) $$\mathrm{Ln}Q=\mathrm{Ln}Q_{0}-K_{1}t.$$

(6) $$\;Q=K_{H}t^{1/2}.$$

(7) $$\mathrm{Ln}Q=n\mathrm{Ln}t+\mathrm{Ln}K_{P}.$$

### 3.6 Interaction between the PA‐N‐2‐HACC‐Cys NPs and mucus

#### 3.6.1 Mucus‐binding assay

Mucin solution was prepared according to a previous report.^67^ Mucin powder from pig stomach was dissolved in ultrapure water (20 mg/mL, pH 4.0), swirled and incubated overnight at 37°C. The mucus suspension was sonicated and purified by centrifugation at 12,000 r/min for 30 min. Mucin solution was co‐incubated with the PA‐N‐2‐HACC NPs, PA‐N‐2‐HACC‐Cys NPs, CUR@PA‐N‐2‐HACC NPs and CUR@PA‐N‐2‐HACC‐Cys NPs, respectively, at 37°C for 4 h (1:1, v/v) and centrifuged at 12,000 r/min for 30 min. Subsequently, the supernatant was transferred to a quartz 96‐well plate and analyzed at 251 nm using a microplate reader (Molecular Devices, SperctraMax190, USA). The mucin concentration in the supernatant was calculated with porcine mucin solution from a standard curve (*y* = 0.2376*x* + 0.0115, *R* ^2^ = 0.999). All the measurements were performed in triplicate. The binding efficiency of mucin was calculated using Formula (8), where *C*_0_ is the initial concentration of mucin, and *C*_S_ is the mucin concentration after incubation with the NPs.

(8) $$\;\text{Binding efficiency}\;\left(\%\right)=\frac{C_{0}-C_{S}}{C_{0}}\times 100.$$

#### 3.6.2 Mucus penetration assay

Mucus‐penetrating ability of the PA‐N‐2‐HACC‐Cys NPs and PA‐N‐2‐HACC NPs was evaluated using a 12‐well transwell plate.^68^ Briefly, 100 μL of the mucin solution was added to each transwell insert, and 1.5 mL of phosphate buffered solution (PBS, pH 7.4) was added to the acceptor chamber. Subsequently, 200 μL of the FITC‐labeled PA‐N‐2‐HACC‐Cys NPs and PA‐N‐2‐HACC NPs were placed onto the mucus layer. The 12‐well transwell plate was incubated at 37°C for 4 h. Next, 200 μL of the samples were removed from the acceptor chamber and placed into a 96‐well plate. The solution was analyzed under a microplate reader for the absorbance at 490 nm. Moreover, the mucus‐penetrating determination methods for PA‐N‐2‐HACC‐Cys NPs and PA‐N‐2‐HACC NPs can be seen in the Supplementary Information. All the measurements were performed in triplicate. The particle penetration of NPs was calculated using Formula (9), where OD_0_ is the initial absorbance of NPs, and OD_S_ is the absorbance of the NPs from the acceptor chamber.

(9) $$\;\text{Particle penetration}\;\left(\%\right)=\frac{{\mathrm{OD}}_{S}}{{\mathrm{OD}}_{0}}\times 100.$$

### 3.7 Cytotoxicity assay

HT29‐MTX and HepG‐2 were cultured in RPMI medium and MEM, respectively, supplemented with 10% fetal bovine serum, 1% (v/v) penicillin, and streptomycin. HT29‐MTX or HepG‐2 were seeded (100 μL) into a 96‐well plate at a cell density of 1 × 10^4^ cells/well. Before that, the free CUR (C400222‐100 g, Aladdin) and NPs were dissolved in the corresponding cell culture medium. The concentration of CUR@PA‐N‐2‐HACC NPs and CUR@PA‐N‐2‐HACC‐Cys NPs was calculated by dividing the set CUR concentration by the corresponding LC. After 24 h, the free CUR, CUR@PA‐N‐2‐HACC‐Cys NPs, or CUR@PA‐N‐2‐HACC NPs within a CUR concentration range of 12.5–200 μg/mL was added to the 96‐well plate containing HepG‐2. In addition, to assess the cytotoxicity of the PA‐N‐2‐HACC‐Cys NPs or PA‐N‐2‐HACC NPs unloaded with CUR, the PA‐N‐2‐HACC‐Cys NPs or PA‐N‐2‐HACC NPs within the concentration range of 0–1000 μg/mL was added to the 96‐well plate containing HT29‐MTX. After co‐incubation for 24 h, the cytotoxicity was analyzed by CCK‐8 assay (Cell Counting Kit‐8, Beyotime).

### 3.8 Cellular uptake

#### 3.8.1 Qualitative analysis of cellular uptake

To investigate the cellular uptake, the cellular uptake of FITC‐labeled PA‐N‐2‐HACC‐Cys NPs and PA‐N‐2‐HACC NPs by HT29‐MTX was observed by CLSM (Olympus, IX83‐FV3000, Japan). Prior to the measurement, these NPs were dissolved in RPMI medium. HT29‐MTX was seeded in the 12‐well plate with 2 × 10^5^ cells/well and incubated overnight. After co‐incubation with 100 μL of the FITC‐labeled PA‐N‐2‐HACC‐Cys NPs or PA‐N‐2‐HACC NPs (1 mg/mL) for 4 h, the cells were washed and stained with 100 nmol/L Lyso‐Tracker Red for 1 h to stain the lysosomes. After fixation with 4% paraformaldehyde, the nuclei were stained with Hoechst 33342 for 10 min. Finally, the cells were washed with PBS (pH = 7.4) and visualized using CLSM.

#### 3.8.2 Quantitative analysis of cellular uptake

Cellular uptake efficiency of the PA‐N‐2‐HACC‐Cys NPs and PA‐N‐2‐HACC NPs was quantified by FCM. HT29‐MTX were seeded in a 12‐well plate at a cell density of 2 × 10^5^ cells/well and incubated overnight. Subsequently, the cells were exposed to 100 μL of the FITC‐labeled PA‐N‐2‐HACC‐Cys NPs or PA‐N‐2‐HACC NPs for 1, 2, and 4 h, and the fluorescence intensity was detected by FCM. Cellular uptake efficiency was calculated using Formula (10), where *P*_S_ is the number of cells that uptake of FITC‐labeled NPs, *P*_0_ is the total number of cells.

(10) $$\;\text{Cellular uptake}\;\left(\%\right)=\frac{P_{S}}{P_{0}}\times 100.$$

#### 3.8.3 Cellular uptake of the NPs loaded with CUR

HT29‐MTX was seeded in the 12‐well plate with 2 × 10^5^ cells/well and incubated overnight. Then, 100 μL of the free CUR, CUR@PA‐N‐2‐HACC NPs and CUR@PA‐N‐2‐HACC‐Cys NPs was added to the HT29‐MTX. The final concentration of CUR in each group was 5, 10, 15, and 20 μg/mL, respectively. The concentration of CUR@PA‐N‐2‐HACC NPs and CUR@PA‐N‐2‐HACC‐Cys NPs was calculated by dividing the set CUR concentration by the corresponding LC. After co‐incubation for prescribed time intervals (2 and 4 h), the cells were cleaned with PBS and lysed by 1% Triton X‐100 at 4°C. After centrifugation at 12,000 r/min for 10 min, 100 μL of the supernatant and 300 μL of methanol were vortexed thoroughly to determine cellular intake of CUR by HPLC. The cellular uptake efficiency was calculated using Formula (11). In addition, after fixation with 4% paraformaldehyde, the HT29‐MTX stained with Hoechst 33342 were observed by CLSM.

(11) $$\text{Cellular uptake}\;\left(\%\right)=\frac{\text{Cellular intake of}\;\mathrm{CUR}}{\text{Total amount of}\;\mathrm{CUR}\;\text{added}}\times 100.$$

### 3.9 Transepithelial transport of the PA‐N‐2‐HACC NPs and PA‐N‐2‐HACC‐Cys NPs

To assess the ability of the PA‐N‐2‐HACC NPs and PA‐N‐2‐HACC‐Cys NPs to transport across mucus and epithelial barriers, HT29‐MTX were seeded in a 12‐well transwell plate and cultured for 21 days to reach a transepithelial electrical resistance (TEER) of 500 Ω cm^2^. Next, the FITC‐labeled PA‐N‐2‐HACC NPs or PA‐N‐2‐HACC‐Cys NPs were added into the donor chamber with a final concentration of 100 μg/mL. In addition, transepithelial transport of the CUR@PA‐N‐2‐HACC‐Cys NPs is described in the Supplementary Information. After incubation for 4 h, 100 μL of the samples were withdrawn from the acceptor chamber, and the fluorescence intensity was measured by a fluorescence spectrophotometer (Hitachi, F‐7000, Japan). All the measurements were performed in triplicate. *P*_app_ was calculated using Formula (12), where *dQ*/*dt* is the flux of FITC‐labeled NPs (μg/s), *C*_0_ is the initial fluorescence intensity of NPs, and *A* is the membrane area (cm^2^) of the transwell.

(12) $$\;P_{\mathrm{app}}\;\left(\%\right)=\frac{\mathrm{dQ}}{\mathrm{dt}}\times \frac{1}{AC_{0}}.$$

Then, the cells were stained with Hoechst 33342 for 10 min and washed with PBS (pH 7.4). Finally, the transwell insert containing the HT29‐MTX monolayer was removed, placed onto a slide, and visualized using CLSM.

### 3.10 In vivo absorption

In vivo transportation of the FITC‐labeled PA‐N‐2‐HACC NPs and FITC‐labeled PA‐N‐2‐HACC‐Cys NPs through the epithelial mucosa was qualitatively analyzed using mice in vivo absorption study.^69^ Male SD rats (6–8 weeks, 230–250 g) were anesthetized by chloral hydrate, and 2‐cm sections of small intestinal loops were ligated at both ends. Then, 100 μL of 1 mg/mL FITC‐labeled PA‐N‐2‐HACC NPs and PA‐N‐2‐HACC‐Cys NPs were administered into different intestinal loops of the same SD rats (*n* = 3). In addition, in vivo absorption of the CUR@PA‐N‐2‐HACC‐Cys NPs is described in the Supplementary Information. After 2 h, the rats were sacrificed, and the loops treated with the FITC‐labeled NPs were withdrawn and washed with PBS (pH = 7.4). After fixation in 4% paraformaldehyde for 4 h and dehydration in 30% sucrose overnight, the loops were embedded in optimal cutting temperature compound and sectioned into 10‐μm slices using a freezing microtome (Leica CM1860, Wetzlar, Germany). Cell nuclei were stained with Hoechst 33342 for 10 min and visualized using CLSM.

### 3.11 Pharmacokinetic study

Nine male SD rats were randomly divided into three groups (*n* = 3). The free CUR, CUR@PA‐N‐2‐HACC NPs, and CUR@PA‐N‐2‐HACC‐Cys NPs were administered by gavage at a dose of 50 mg CUR per kilogram body weight. Moreover, 500 μL of blood was taken before administration, and 300 μL of blood was taken from the heart at 0.25, 0.5, 1, 2, 4, 6, 9, 12, and 24 h after administration. The plasma samples were centrifuged at 3000 r/min for 10 min. Subsequently, 100 μL of plasma was mixed with 10 μL methanol containing internal standard (rheum emodin, 20 μg/mL). After vortexing for 3 min, 250 μL ethyl acetate was added, and the mixture was centrifuged at 3000 r/min for 10 min. The extraction residue was reconstituted in 100 μL of mobile phase and determined by HPLC. The mobile phase was a mixture of acetonitrile: water (containing 2% acetic acid) (52:48, v/v), the flow rate was 1 mL/min, and the detection wavelength was 426 nm. Pharmacokinetic parameters were calculated using PK SOLVER for the noncompartmental model.

### 3.12 Efficacy of the CUR@PA‐N‐2‐HACC‐Cys NPs on colitis mice

Male BALB/c mice (6–8 weeks, 20–25 g) were randomly divided into five groups with eight mice in each group after 1 week of acclimation. Except for the normal control group, the remaining four groups were given 3% DSS instead of ordinary drinking water for 7 days, during which they were fed normally to establish the mice colitis model. It is advisable for human body to take CUR 180–500 mg per day.^70^ Based on the conversion of doses given to humans and animals, the oral dose of CUR for mice is 50 mg CUR per kilogram body weight. The treatment groups were set as follows: (1) control group: ordinary drinking water for 7 days, followed by daily oral administration of PBS for 7 days; (2) DSS + PBS group: 3% DSS for 7 days, followed by daily oral administration of PBS for 7 days; (3) DSS + PA‐N‐2‐HACC‐Cys NPs group: 3% DSS for 7 days, followed by daily oral administration of PA‐N‐2‐HACC‐Cys NPs for 7 days; (4) DSS + CUR group: 3% DSS for 7 days, followed by daily oral administration of free CUR for 7 days at a dose of 50 mg CUR per kilogram body weight; (5) DSS + CUR@PA‐N‐2‐HACC NPs group: 3% DSS for 7 days, followed by daily oral administration of the CUR@PA‐N‐2‐HACC NPs for 7 days, in which the dose of CUR contained in the CUR@PA‐N‐2‐HACC NPs was 50 mg/kg; (6) DSS + CUR@PA‐N‐2‐HACC‐Cys NPs group: 3% DSS for 7 days, followed by daily oral administration of CUR@PA‐N‐2‐HACC NPs for 7 days, in which the dose of CUR contained in the CUR@PA‐N‐2‐HACC‐Cys NPs was 50 mg/kg. The body weight of the mice was recorded every day, and DAI was measured to assess the severity of the colitis according to body weight loss, character of stool, and the degree of hematochezia (Table 3).

After treatment with different formulas, the mice were sacrificed after anesthesia, the colon length was measured, and the colon tissue was fixed with 10% formalin and stained with hematoxylin and eosin (H&E) for pathological examination. To count colonic goblet cells, fixed colonic tissues were also stained using Alcian blue. The slides of colon tissue were immunostained by a primary mouse ZO‐1 antibody and mouse Claudin‐1 antibody (Elabscience, China), and combined with a classical streptavidin–biotin–peroxidase detection system for immunohistochemical analysis.

In addition, a portion of collected colon tissue (50 mg) was homogenized with RIPA lysis buffer (Thermo Fisher, Shanghai, China) to extract total proteins. The homogenate was centrifuged at 12,000 r/min for 15 min at 4°C. The protein content of supernatant was quantified using a bicinchoninic acid (BCA) protein assay kit (Thermo Fisher, Shanghai, China). The concentration of pro‐inflammatory cytokines such as TNF‐α and IL‐1β was measured by ELISA kit (Enzyme‐linked Biotechnology Co., Ltd., China).

### 3.13 Statistical analysis

All the experimental data were represented as mean ± standard deviation (SD), and analyzed using one‐way analysis of variance (ANOVA) (GraphPadPrism software Inc., USA). *p* < 0.05 was considered statistically significant.

## 4 CONCLUSIONS

Novel PA‐ and Cys‐functionalized NPs were synthesized from N‐2‐HACC modified with PA and Cys. The NPs could prevent CUR from degradation and prolong the retention time in the gastrointestinal tract, penetrate mucus, and open the TJ between epithelial cells. The PA‐N‐2‐HACC‐Cys NPs exhibited excellent mucoadhesion, sustained slow release, and permeability, indicating that the NPs could overcome both mucus and epithelial barriers and significantly improve the hydrophilicity, stability, and bioavailability of drugs for oral administration. In vivo experiments confirmed that oral administration of the CUR@PA‐N‐2‐HACC‐Cys NPs significantly ameliorated DSS‐induced colitis, with an efficacy superior to that of the free CUR and CUR@PA‐N‐2‐HACC NPs. Therefore, N‐2‐HACC NPs modified with PA and Cys showed significant competitive advantages in multifunctional drug delivery vectors, providing a new perspective for expanding the potential application of hydrophobic drug.

## AUTHOR CONTRIBUTIONS

**Yinzhuo Xie:** Conceptualization (lead); data curation (lead); formal analysis (equal); investigation (equal); methodology (lead); validation (lead); writing – original draft (lead); writing – review and editing (lead). **Zheng Jin:** Conceptualization (equal); data curation (equal); formal analysis (equal); investigation (equal); methodology (equal); validation (equal); writing – review and editing (equal). **Da Ma:** Data curation (equal); methodology (equal). **Tan Hui Yin:** Formal analysis (equal); writing – review and editing (equal). **Kai Zhao:** Conceptualization (lead); data curation (lead); formal analysis (equal); funding acquisition (lead); investigation (lead); methodology (lead); project administration (lead); validation (equal); writing – original draft (equal); writing – review and editing (lead).

## CONFLICT OF INTEREST STATEMENT

The authors declare no competing financial interest.

### PEER REVIEW

The peer review history for this article is available at https://www.webofscience.com/api/gateway/wos/peer-review/10.1002/btm2.10510.

## Supporting information


**Data S1:** Supporting Information

## Data Availability

The main data supporting the results in this study are available within the paper and its supporting information. Additional data related to this work are available for research purposes from the corresponding author on reasonable request.
