# Supplementary material for: Palmitic acid‐ and cysteine‐functionalized nanoparticles overcome mucus and epithelial barrier for oral delivery of drug
Source: Bioeng Transl Med. 2023 Mar 23;8(3):e10510. doi: 10.1002/btm2.10510 (PMC10189451; doi:10.1002/btm2.10510)
Supplement: Supplementary file 1 — Data S1: Supporting Information [file BTM2-8-e10510-s001.docx]

**Palmitic acid- and cysteine-functionalized nanoparticles overcome mucus and epithelial barrier** **for oral delivery of drug**

*Yinzhuo Xie ^1, 2^, Zheng Jin ^1, 2^, Da Ma ^3^, Tan Hui Yin ^4^, Kai Zhao ^1, 2, *^*

*^1^ Institute of Nanobiomaterials and Immunology, Institute for Advanced Studies, Taizhou University, Taizhou 318000, China*

*^2^ Zhejiang Provincial Key Laboratory of Plant Evolutionary Ecology and Conservation, School of Life Science, Taizhou University, Taizhou 318000, China*

*^3^ School of Pharmaceutical and Materials Engineering & Institute for Advanced Studies, Taizhou Univerisity, Taizhou 318000, China*

*^4^ Tunku Abdul Rahman University of Management and Technology, Jalan Genting Kelang, Kuala Lumpur 53300, Malaysia*

**Running title:** Functionalized nanoparticles for oral delivery

** Correspondence: Kai Zhao, Tel.: +86 576 88660338, E-mail: zybin395@126.com.*

**1. Materials and methods**

**1.1. Characterization of the PA-N-2-HACC-Cys**

***1.1.1. Structural characterization of the PA-N-2-HACC-Cys***

The structures of the PA-N-2-HACC-Cys were recorded within the range of 4,000 cm^−1^ to 400 cm^−1^ at a scanning rate of 4 cm^−1^ by FT-IR spectroscopic (Nicolet, IS10, USA) and ^1^H-nuclear magnetic resonance (^1^H-NMR) spectra (Bruker, AVANCE III HD 400, Germany).

***1.1.2. Quantification of thiol contents***

The content of thiol groups on the PA-N-2-HACC-Cys was determined using Ellman’s reagent.^1^ Briefly, 10 mg of the PA-N-2-HACC-Cys was dissolved in 0.5 mol/L of phosphate-buffered saline (PBS, pH 8.0), and 1 mL of the PA-N-2-HACC-Cys solutions (0.25 mg/mL) was reacted with 2 mL of Ellman’s PBS solution (3.0 mg/mL) at room temperature in the dark for 2 h. The absorbance of the reaction solution was taken spectrophotometrically at a wavelength of OD_420_. The number of thiol groups on the PA-N-2-HACC-Cys was calculated from a standard curve with an Cys solution (R^2^=0.9996).

**1.2. Preparation of the FITC-labeled polymer NPs**

FITC-labeled the PA-N-2-HACC-Cys NPs and PA-N-2-HACC NPs were prepared by phacoemulsification. 1 mL of FITC in dichloromethane was added dropwise into the polymer solution. The content of FITC was 0.1% of the amount of carrier polymer, and the preparation method was the same as that of CUR@PA-N-2-HACC-Cys NPs.

***1.2.1. In vitro*** ***leakage of the*** **FITC-labeled polymer NPs**

To investigate the leakage of FITC-labeled polymer NPs *in vitro*, the dialysis bag was filled with 4 mL of the FITC-labeled polymer NPs solution (1 mg/mL) and immersed in 40 mL of the phosphate buffered solution (pH=7.4) at 37 ℃. During this period, 400 µL of the release medium was removed at different time (0.5, 1, 2, 4, 6, 8, 10, 12, and 24 h) to measure the FITC concentration under a UV-visible spectrophotometer (Hitachi, U-5100, Japan) for absorption at 490 nm. The standard curve was prepared in the concentration range of 10^-3^–0.05 mg/mL (y=0.0282x+0.0284) (R^2^=0.999). All the measurements were performed in triplicate. The total concentration of FITC and the concentration in the released sample were determined, and the cumulative release of FITC was calculated.

**1.3.** **Interaction between the CUR@PA-N-2-HACC-Cys NPs and mucus**

***1.3.1. Mucus penetration assay***

Mucus-penetrating ability of the CUR@PA-N-2-HACC-Cys NPs and CUR@PA-N-2-HACC NPs was evaluated using a 12-well transwell plate. Briefly, 100 μL of the mucin solution was added to each transwell insert, and 1.5 mL of phosphate buffered solution (PBS, pH=7.4) was added to the acceptor chamber. Subsequently, 200 μL of the CUR@PA-N-2-HACC-Cys NPs or CUR@PA-N-2-HACC NPs (1 mg/mL) were placed onto the mucus layer. The 12-well transwell plate was incubated at 37 ℃ for 4 h. Next, 200 μL of the samples were removed from the acceptor chamber, followed by adding methanol to 4 mL, ultrasonic demulsification, centrifugation to take the supernatant, using HPLC to determine the content of CUR. The particle penetration of NPs was calculated using formula (1), where A_0_ is the initial CUR concentration of NPs, and A_S_ is the CUR concentration of NPs from the acceptor chamber. All the measurements were performed in triplicate.

$$\text{ Particle penetration}\text{ }\text{(\%)}\text{ }\text{= }\frac{\text{A}_{\text{S}}}{\text{A}_{\text{0}}}\text{ × }\text{100}\text{ }\text{(1)}$$

**1.4.** **Cellular uptake of the CUR@PA-N-2-HACC-Cys NPs**

HT29-MTX was seeded in the 12-well plate with 2×10^5^ cells/well and incubated overnight. Then, 100 μL of the free CUR, CUR@PA-N-2-HACC NPs and CUR@PA-N-2-HACC-Cys NPs was added to the HT29-MTX. The final concentration of CUR in each group was 5, 10, 15, 20 μg/mL, respectively. After co-incubation for prescribed time intervals (2 and 4 h), the cells were cleaned with PBS and lysed by 1% Triton X-100 at 4 ℃. After centrifugation at 12,000 r/min for 10 min, 100 µL of supernatant and 300 µL of methanol were vortexed thoroughly to determine CUR content by HPLC. In addition, the HT29-MTX stained with Hoechst 33342 were observed by CLSM.

**1.5.** **Transepithelial transport of the CUR@PA-N-2-HACC-Cys NPs**

Transepithelial transport study of NPs was carried out on the HT29-MTX monolayers seeded on a 12-well transwell insert fitted with polycarbonate membranes. Prior to the study, TEER reached Ω·cm^2^, followed by the addition of CUR@PA-N-2-HACC NPs or CUR@PA-N-2-HACC-Cys NPs within the CUR concentration of 200 μg/mL. After incubation for 4 h, 100 μL of samples were withdrawn from the acceptor chambers. The amount of transported CUR@PA-N-2-HACC NPs was measured by HPLC. The *P*_app_ was calculated using formula (2), where dQ/dt is the CUR flux of NPs from the acceptor chamber (μg/s), C_0_ is the initial CUR concentration of NPs, and A is the membrane area (cm^2^) of the transwell.

$$\text{ }\text{P}_{\text{app}}\text{ (\%)}\text{ }\text{= }\frac{\text{dQ}}{\text{dt}}\text{ × }\frac{\text{1}}{\text{A}\text{C}_{\text{0}}}\text{ }\text{ }\text{ }\text{ }\text{ }\text{ }\text{ }\text{(2)}$$

**1.6. *In vivo* absorption**

All the animal care and experimental protocols were in accordance with the “National Research Council's Guide for the Care and Use of Laboratory Animals”. Male SD rats (6-8 weeks, 230-250 g) were anesthetized by chloral hydrate, and 2 cm sections of small intestinal loops were ligated at both ends. Then, 100 µL of the free CUR, CUR@PA-N-2-HACC-Cys NPs and CUR@PA-N-2-HACC NPs within the CUR concentration of 200 μg/mL was administered into different intestinal loops of the same SD rats (n=3). After 2 h, the rats were sacrificed, and the loops treated with the various CUR formulations were withdrawn and washed with PBS (pH=7.4). After fixation in 4% paraformaldehyde for 4 h and dehydration in 30% sucrose overnight, the loops were embedded in optimal cutting temperature compound and sectioned into 10-μm slices using a freezing microtome (Leica CM1860, Wetzlar, Germany). Cell nuclei were stained with Hoechst 33342 for 10 min and visualized using CLSM.

**2. Results**

**2.1. FT-IR analysis**

The PA-N-2-HACC-Cys retained the characteristic peaks of N-2-HACC and PA-N-2-HACC, and also exhibited new peaks. Figure. S1a shows that the peaks corresponding to the methyl protons (−CH_3_ and −CH_2_−) stretching bands were observed at 2,920 cm^−1^ and 2,849 cm^−1^.^2, 3^ The peak at 1,740 cm^−1^ for ester bond stretching vibration and the peak corresponding to CONH stretching vibration at 1,690 cm^−1^ (amide I) were not weakened.^2^ Moreover, a weak signal of the thiol group at 2,520 cm^−1^ also confirmed the introduction of Cys with the −SH into the PA-N-2-HACC.

**2.2. ^1^H-NMR analysis**

Figure. S1b showed that ^1^H-NMR spectra of the PA-N-2-HACC-Cys presented the peaks at δ=0.88 and δ=1.24 ppm were assigned to the methyl protons of PA (−CH_3_ and −CH_2_−).^2, 3^ The protons in the thiol group could easily be replaced by deuterohydrogen in deuteroxide (D_2_O) used for NMR sample preparation, making it undetectable in the NMR spectrum.^4^ Nevertheless, the signal at δ=2.95 ppm for methylene protons adjacent to the mercaptan group (−S−CH_2_) was observed, which could be taken as evidence of the presence of the thiol group covalently linked to the PA-N-2-HACC.^5-8^ Both the ^1^H-NMR and FT-IR spectra indicated that the PA-N-2-HACC had excellent conjugation with Cys.


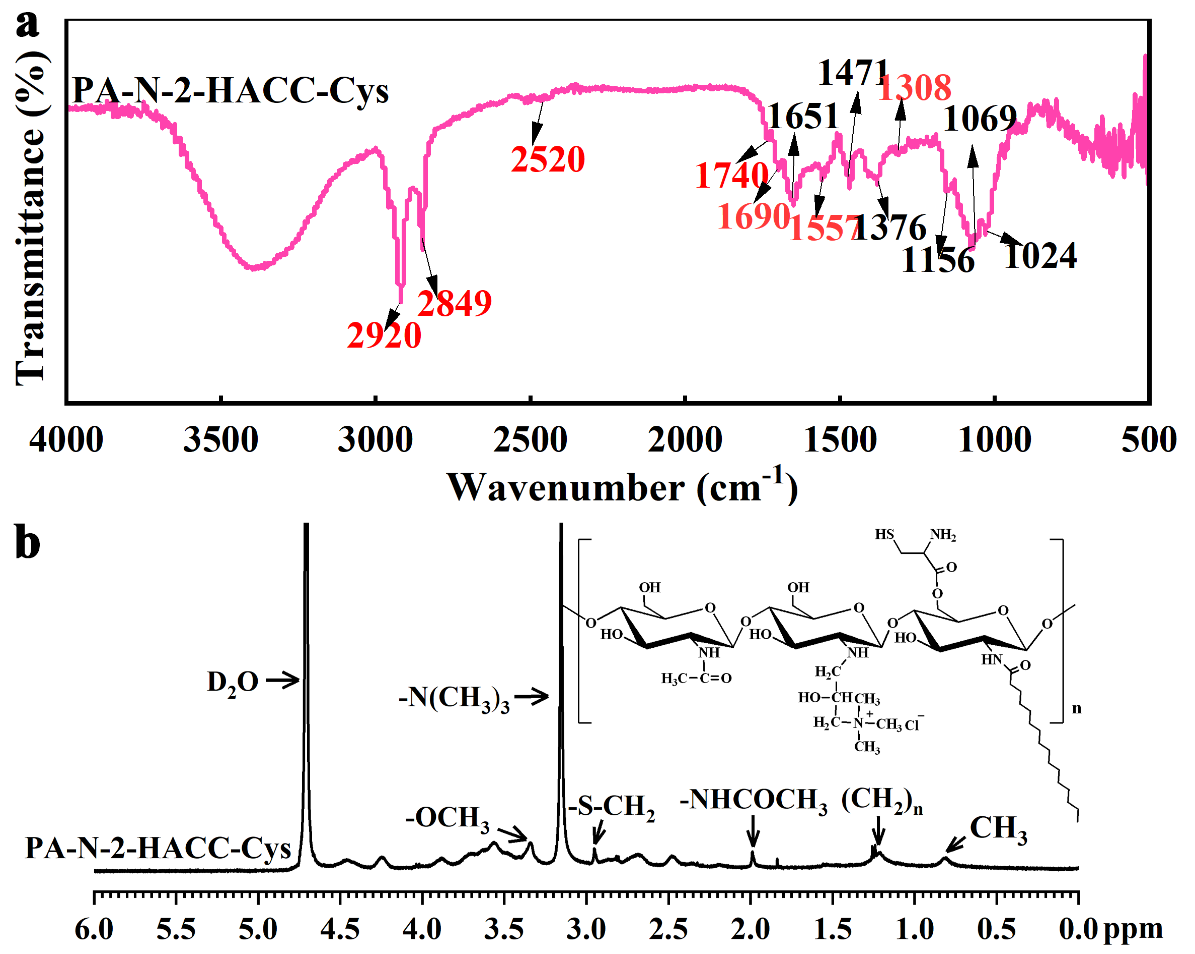


Supplementary Figure 1. FT-IR spectra (a) and ^1^H-NMR spectra (b) of the CUR@PA-N-2-HACC-Cys NPs.

**2.3. Number of thiol groups on the PA-N-2-HACC-Cys**

The number of free thiol groups on the PA-N-2-HACC-Cys was determined by comparing the slopes using the linear regression. According to the standard curve, the number of thiol groups attached to PA-N-2-HACC-Cys was 274.125 µmol/g.

**2.4. Characterization of NPs**

By comparing the different NPs (Table S1), we found that the particle size of the NPs was larger due to CUR encapsulation; and the particle size of the PA-N-2-HACC-Cys NPs was smaller than that of the PA-N-2-HACC NPs, which might be due to the tighter internal structure of NPs caused by the introduction of Cys. In addition, compared with the CUR@PA-N-2-HACC NPs, EE and LC of the CUR@PA-N-2-HACC-Cys NPs were increased.

**Table S1** Characteristics of CUR-loaded NPs (n=3)

| **Samples** | **Particle size(nm)** | **Zeta (mV)** | **PDI** | **EE (%)** | **LC (%)** |
| --- | --- | --- | --- | --- | --- |
| CUR@PA-N-2-HACC-Cys NPs | 213.2±4.78 | +44.1±0.26 | 0.20±0.01 | 85.10±1.43 | 9.41±0.73 |
| CUR@PA-N-2-HACC NPs | 264.5±4.11 | +43.2±1.82 | 0.206±0.017 | 75.43±1.25 | 6.81±0.16 |
| PA-N-2-HACC-Cys NPs | 193.1±5.68 | 41.6±0.51 | 0.264±0.028 | —— | —— |
| PA-N-2-HACC NPs | 231.6±9.24 | +37.0±2.12 | 0.21±0.013 | —— | —— |

**2.5.** ***In vitro* leakage of the FITC-labeled polymer NPs**

The results of FITC-labeled NPs leakage *in vitro* are shown in Figure. S2. The *in vitro* leakage of the FITC-labeled PA-N-2-HACC-Cys NPs and PA-N-2-HACC NPs was 0.1% and 0.55% at 2 h, and 5% and 3% at 24 h, respectively, indicating that the fluorescent substance can be stably encapsulated in polymer NPs. The leakage is very small and slow, which can be used for NPs tracking *in vitro*.


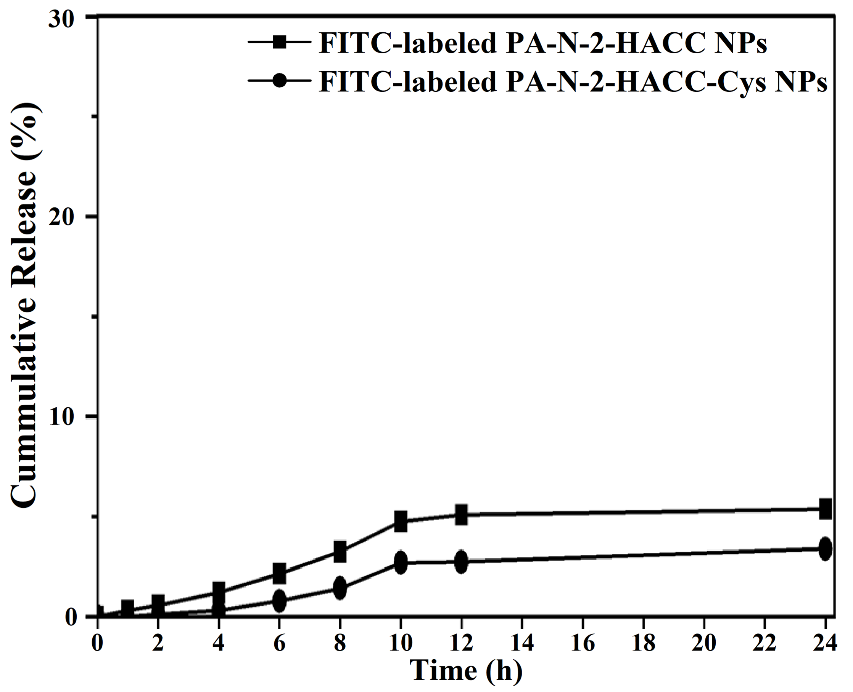


Supplementary Figure 2. Leakage of FITC-labeled PA-N-2-HACC-Cys NPs and PA-N-2-HACC NPs in PBS (pH=7.4).

**2.6. Transepithelial transport of the CUR@PA-N-2-HACC-Cys NPs**

More importantly, SEM images also indicated that the CUR@PA-N-2-HACC-Cys NPs showed more complete morphology in the lower chamber after incubation with HT29-MTX (Figure. S3). The result confirmed that the transepithelial transport efficiency of the CUR@PA-N-2-HACC-Cys NPs was much stronger than that of the CUR@PA-N-2-HACC NPs.


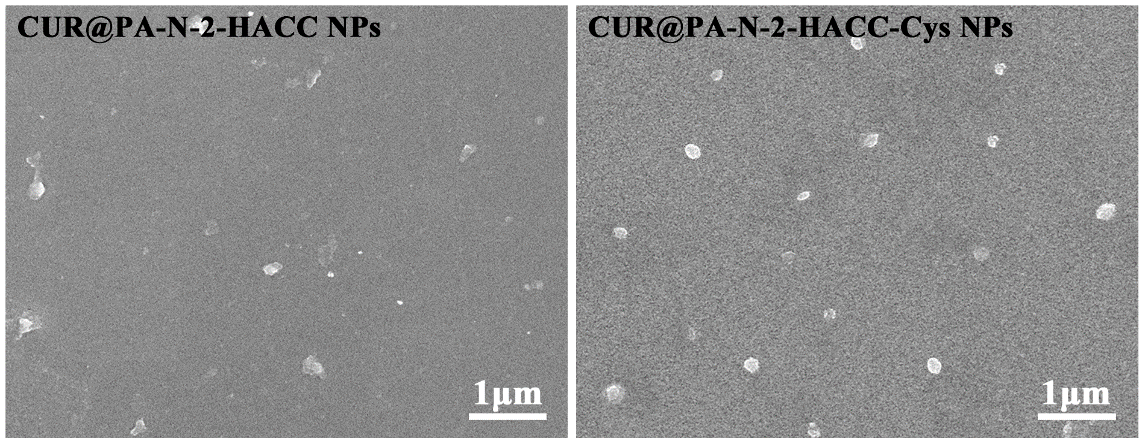


Supplementary Figure 3. SEM image of the CUR@PA-N-2-HACC NPs and CUR@PA-N-2-HACC-Cys NPs in the lower chamber after incubation with HT29-MTX for 4 h. Scale bars=1 μm.

**References**

1. Zhou SR, Deng HL, Zhang Y, et al. Thiolated nanoparticles overcome the mucus barrier and epithelial barrier for oral delivery of insulin. Mol Pharm. 2020; 17(1):239-250. https://doi:10.1021/acs.molpharmaceut.9b00971.

2. Xie YZ, Gong XC, Jin Z, Xu W, Zhao K. Curcumin encapsulation in self-assembled nanoparticles based on amphiphilic palmitic acid-grafted-quaternized chitosan with enhanced cytotoxic, antimicrobial and antioxidant properties. Int J Biol Macromol. 2022; 222:2855-2867.

3. Ramalingam P, Ko YT. Improved oral delivery of resveratrol from N-trimethyl chitosan-g-palmitic acid surface-modified solid lipid nanoparticles. Colloids Surf B Biointerfaces. 2016; 139:52-61. https://doi:10.1016/j.colsurfb.2015.11.050.

4. Medeiros Borsagli FGL, Carvalho IC, Mansur HS. Amino acid-grafted and N-acylated chitosan thiomers: Construction of 3D bio-scaffolds for potential cartilage repair applications. Int J Biol Macromol. 2018; 114:270-282. https://doi:10.1016/j.ijbiomac.2018.03.133.

5. Liu DD, Li JY, Pan H, et al. Potential advantages of a novel chitosan-N-acetylcysteine surface modified nanostructured lipid carrier on the performance of ophthalmic delivery of curcumin. Sci Rep. 2016; 6:28796. https://doi.org/10.1038/srep28796.

6. Rajawat GS, Shinde UA, Nair HA. Chitosan-N-acetyl cysteine microspheres for ocular delivery of acyclovir: Synthesis and in vitro/in vivo evaluation. J Drug Deliv Sci Technol. 2016; 35:333-342. https://doi.org/10.1016/j.jddst.2016.08.006.

7. Liu XJ, Yu B, Huang QL, et al. *In vitro* BMP-2 peptide release from thiolated chitosan based hydrogel. Int J Biol Macromol. 2016; 93:314-321. https://doi.org/10.1016/j.ijbiomac.2016.08.048.

8. Li JY, Liu DD, Tan GX, et al. A comparative study on the efficiency of chitosan-N-acetylcysteine, chitosan oligosaccharides or carboxymethyl chitosan surface modified nanostructured lipid carrier for ophthalmic delivery of curcumin. Carbohydr Polym. 2016; 146:435-444. https://doi.org/10.1016/j.carbpol.2016.03.079.
